# High Confidence Prediction of Essential Genes in *Burkholderia Cenocepacia*

**DOI:** 10.1371/journal.pone.0040064

**Published:** 2012-06-29

**Authors:** Mario Juhas, Manuel Stark, Christian von Mering, Puthapoom Lumjiaktase, Derrick W. Crook, Miguel A. Valvano, Leo Eberl

**Affiliations:** 1 Department of Microbiology, Institute of Plant Biology, University of Zurich, Zurich, Switzerland; 2 Institute of Molecular Life Sciences and Swiss Institute of Bioinformatics, University of Zurich, Zurich, Switzerland; 3 Mahidol University, Salaya, Nakhon Pathom, Thailand; 4 Nuffield Department of Clinical Laboratory Sciences, John Radcliffe Hospital, University of Oxford, Oxford, United Kingdom; 5 Department of Microbiology and Immunology, University of Western Ontario, London, Ontario, Canada; Louisiana State University and A & M College, United States of America

## Abstract

**Background:**

Essential genes are absolutely required for the survival of an organism. The identification of essential genes, besides being one of the most fundamental questions in biology, is also of interest for the emerging science of synthetic biology and for the development of novel antimicrobials. New antimicrobial therapies are desperately needed to treat multidrug-resistant pathogens, such as members of the *Burkholderia cepacia* complex.

**Methodology/Principal Findings:**

We hypothesize that essential genes may be highly conserved within a group of evolutionary closely related organisms. Using a bioinformatics approach we determined that the core genome of the order *Burkholderiales* consists of 649 genes. All but two of these identified genes were located on chromosome 1 of *Burkholderia cenocepacia*. Although many of the 649 core genes of *Burkholderiales* have been shown to be essential in other bacteria, we were also able to identify a number of novel essential genes present mainly, or exclusively, within this order. The essentiality of some of the core genes, including the known essential genes *infB*, *gyrB*, *ubiB*, and *valS*, as well as the so far uncharacterized genes *BCAL1882*, *BCAL2769*, *BCAL3142* and *BCAL3369* has been confirmed experimentally in *B. cenocepacia*.

**Conclusions/Significance:**

We report on the identification of essential genes using a novel bioinformatics strategy and provide bioinformatics and experimental evidence that the large majority of the identified genes are indeed essential. The essential genes identified here may represent valuable targets for the development of novel antimicrobials and their detailed study may shed new light on the functions required to support life.

## Introduction

Essential genes, considered to be the foundation of life, are absolutely required for the survival of an organism. Identification of the minimal set of genes needed to sustain a life form is expected to contribute greatly to our understanding of life at its simplest and fundamental level. Determination of a minimal genome not only contributes to basic biology but also plays an important role in the emerging field of synthetic biology, whose main goal is to synthesize living cells with rewired circuits to fulfil designed properties [1]–[3]. Furthermore, due to their indispensability for bacterial cell survival, essential genes also represent promising targets of novel antimicrobials [4]. Several experimental and computational approaches have been employed for the identification of genes that are considered to be essential for cell viability [5]–[24]. Genes involved in DNA replication, transcription and translation and membrane biogenesis have been found in all minimal genome analyses and are therefore considered universally essential. However, the exact composition of the minimal genome is still unknown for most lineages [4].

*Burkholderiales* have come to the focus of the minimal genome research for two major reasons. First, many species of this order harbour more than one chromosome and have very large genomes, which make them biologically interesting. Second, the genome of many strains has been sequenced, thus allowing meaningful comparisons. The genus *Burkholderia* comprises more than 50 species, which differ not only in the composition of their genomes but also in their lifestyles [25]–[27]. It includes plant symbionts as well as bacteria involved in degradation of pollutants and clinically important opportunistic human pathogens [28], [29]. *Burkholderia mallei* and *Burkholderia pseudomallei*, causing glanders and melliodosis, respectively, are considered agents of bio-terrorism due to their low infectious doses and high fatality rate in human infections. *Burkholderia cenocepacia* is an important pathogen of cystic fibrosis patients that can cause a rapid decline in patient’s health due to necrotizing pneumonia and septicaemia resulting in early death known as ‘cepacia syndrome’ [29]. In addition to harbouring various virulence traits, pathogenic *Burkholderia* strains are also highly resistant to a wide variety of antibiotics and thus novel antimicrobials targeting this group of microorganisms are urgently needed [30], [31]. Determination of the *Burkholderia* minimal genome could help identify novel targets for the development of antimicrobials.

Here, we show that the core genome of the order *Burkholderiales* consists of 649 genes the majority of which are homologous to essential genes identified in other species. By constructing conditional knock-out mutants in the model organism *B. cenocepacia* H111 we provide experimental evidence of the essentiality of some of these identified genes for *B. cenocepacia*, including four genes of unknown function. Two of these uncharacterized genes belong to a subset of 84 genes identified in our study, which have not yet been described to be essential in another organism. Furthermore we show that the vast majority of essential genes in *B. cenocepacia* are located chromosome 1.

## Results and Discussion

### Computation of the Core Genome of the Order *Burkholderiales*

**Figure 1:**
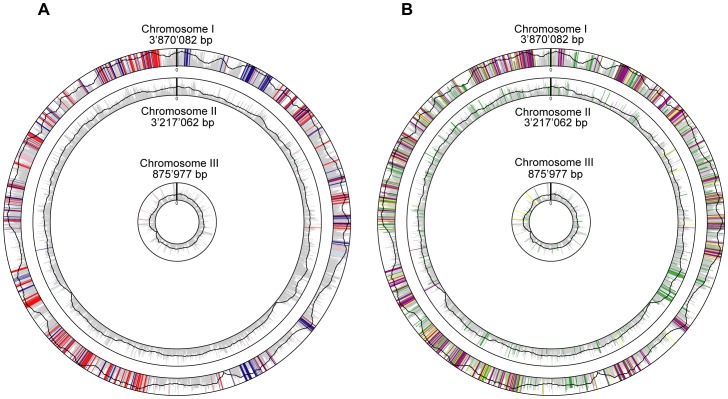
Chromosome 1 harbours most of the core genome. A) Schematic view of chromosomes 1–3 of *Burkholderia cenocepacia* J2315. The 649 genes belonging to our core genome are indicated by blue and red bars (positive or negative gene direction respectively). Core genes with homologues within the core genome are printed in light blue and rose. Other genes, which are less conserved in respect of presence within the *Burkholderiales* are indicated by grey bars (the height indicates the degree of conservation). The black graph also indicates the degree of conservation. 636 core genes belong to chromosome 1. Out of the remaining 13 genes, only two that are located on chromosome 2 are singletons (do not have other homologues within the genome). B) 454 core genes have homologues in the DEG database and are thus predicted to be essential (violet). Our core genome contains 195 genes without clear orthologues in the DEG database (yellow) 111 of these genes do show weak homology to DEG. The other 84 are potentially novel essential genes. 574 of *B. cenocepacia* J2315 homologues to the DEG database do not belong to our core genome (green).

The pan-genome of the genus *Burkholderia*, which represents all genes potentially present in a genome of this bacterial genus, currently consists of approximately 50 000 genes [28]. This high number reflects the enormous metabolic diversity of the genus, which appears to be a consequence of the horizontally acquired genetic elements [32]–[36]. Previous work has suggested that the core genome of the genus *Burkholderia*, i.e. those genes that are highly conserved across all *Burkholderia* genomes, consists of only a few hundred open reading frames [28]. To extend this study and to generate a list of putative essential genes, we employed a bioinformatics approach described in the Materials and Methods section to determine the core genome of the order *Burkholderiales*. Our analysis revealed 610 orthologous groups that are present in all 51 *Burkholderiales* genomes (Table S1) which are currently available in the STRING9 database [37]. Therefore we consider these 610 orthologous groups to represent the core genome of the order *Burkholderiales*. In our reference strain selected for the genomic analysis, *Burkholderia cenocepacia* J2315, these 610 orthologous groups correspond to 649 genes (Figure 1) (Table S2). Paralogous genes, most of which are highly homologous and thus have only recently been duplicated were included in our analysis as they could still be interesting targets for the development of antimicrobial compounds, which likely would inhibit all the closely related paralogs simultaneously.

### Identification of Essential Genes and Operons in *B. cenocepacia*

While bacterial strains of the same genus often differ greatly in the composition of their genomes they usually share a set of well-conserved essential genes [15], [20]. We therefore reasoned that the core genome identified should mainly consist of genes that are essential for growth and survival of members of the *Burkholderiales*. To test this hypothesis, we first searched the *Burkholderiales* core genome for essential genes previously identified in other bacterial species, namely *Pseudomonas aeruginosa* [14], *E. coli* [38] and *B. subtilis* [8]. 59, 41, and 62 genes of the core genome were found among essential genes of *P. aeruginosa*, *E. coli* and *B. subtilis*, respectively, using the stringent minimum cut-offs (Materials and Methods) for pairwise comparison in our study. 101 genes of the *Burkholderiales* core genome were found to be essential in at least one of the three bacteria. These initial searches supported the idea that the *Burkholderiales* core genome harbors a number of essential genes. To further extend our study we searched the *Burkholderiales* core genome for homologues of essential genes in the database of essential genes (DEG) [39], [40], which contains 12297 genes identified in a number of prokaryotes and eukaryotes. This revealed that the vast majority of the *Burkholderiales* core genes (454 out of 649) are homologous to genes previously shown to be essential in other organisms (Figure 1, Table S3). Statistical analysis of the DEG homologues showed that they are significantly enriched in our core genome. This is the case both for genes on chromosome 1 and chromosome 2 (p-values <0.001 and <0.002 respectively). This result further emphasizes the importance of our core genome for the function of the cell.

To verify the essentiality of genes in the core genome of *B. cenocepacia*, we decided to generate conditional knock-down mutants. One way to generate such mutants is to replace the native promoter of an essential gene or operon with one that can be stringently controlled (Juhas et al, unpublished). An advantage of the promoter-replacement systems is that the native open reading frame of the gene is maintained [41]. Based on the *E. coli* rhamnose-inducible promoter P*_rhaB_* such knock-down systems have been developed previously for the identification of essential genes and operons in *B. cenocepacia* [42]–[44]. In our study we used plasmid pSC200, which allows the delivery of a rhamnose-inducible promoter upstream of genes of interest (Figure S1). In this approach, approximately 300 bp fragments spanning the 5′ region of a targeted gene were cloned into pSC200 and the resulting recombinant plasmids were subsequently transferred into the model strain *B. cenocepacia* H111 by triparental mating. *Burkholderia* conditional rhamnose-dependent mutants are generated by homologous recombination where the native promoters of targeted genes are replaced for the rhamnose-inducible promoter introduced by the plasmid [43]. In the constructed conditional mutant strains, the investigated gene is located downstream of the rhamnose promoter, and thus its expression is stringently controlled by the amount of rhamnose in the growth medium (Figure S1). As a proof of principle we have chosen six singleton genes (with no paralogs in the *B. cenocepacia* J2315 genome) that were previously demonstrated to be essential in another organism, namely: *infB, gyrB, ubiB, valS*, *BCAL3142* and *BCAL3369* (Figure S2).

Gene essentiality is condition dependent. For example, while the referred DEG database lists 1617 E. coli MG1655 genes as essential, other experimentally more rigorous studies list only around 300 essential genes [12]. In our analysis we have investigated essentiality of selected genes in LB medium supplemented with either 0.5% rhamnose (permissive condition) or 0.5% glucose (non-permissive condition) as described in the Materials and Methods section. The growth of *B. cenocepacia* H111 strain in permissive and non-permissive conditions in LB medium was unaltered, thus showing that the presence of rhamnose or glucose in the medium does not have any effect on the growth of *B. cenocepacia* H111 wild type strain (Figure S3). To control for possible errors in the conditional mutagenesis and complementation strategy, two additional mutants in non-essential genes, which were not part of the core genome, were constructed, H111*engA* and H111*2430*. H111*2430* conditional mutant grew both in the presence of rhamnose and glucose (Figure S3), confirming that our approach is suitable for the identification of essential genes in *B. cenocepacia*. H111*engA* conditional mutant grew in the presence of rhamnose but was unable to grow in the presence of glucose (Figure S3). Expectedly, complementation *in trans* did not restore the ability of the H111*engA* to grow in glucose (Figure S3), showing that the growth deficiency of H111*engA* was a result of polar effects of downstream essential genes and not of the essentiality of *engA*.

**Figure 2:**
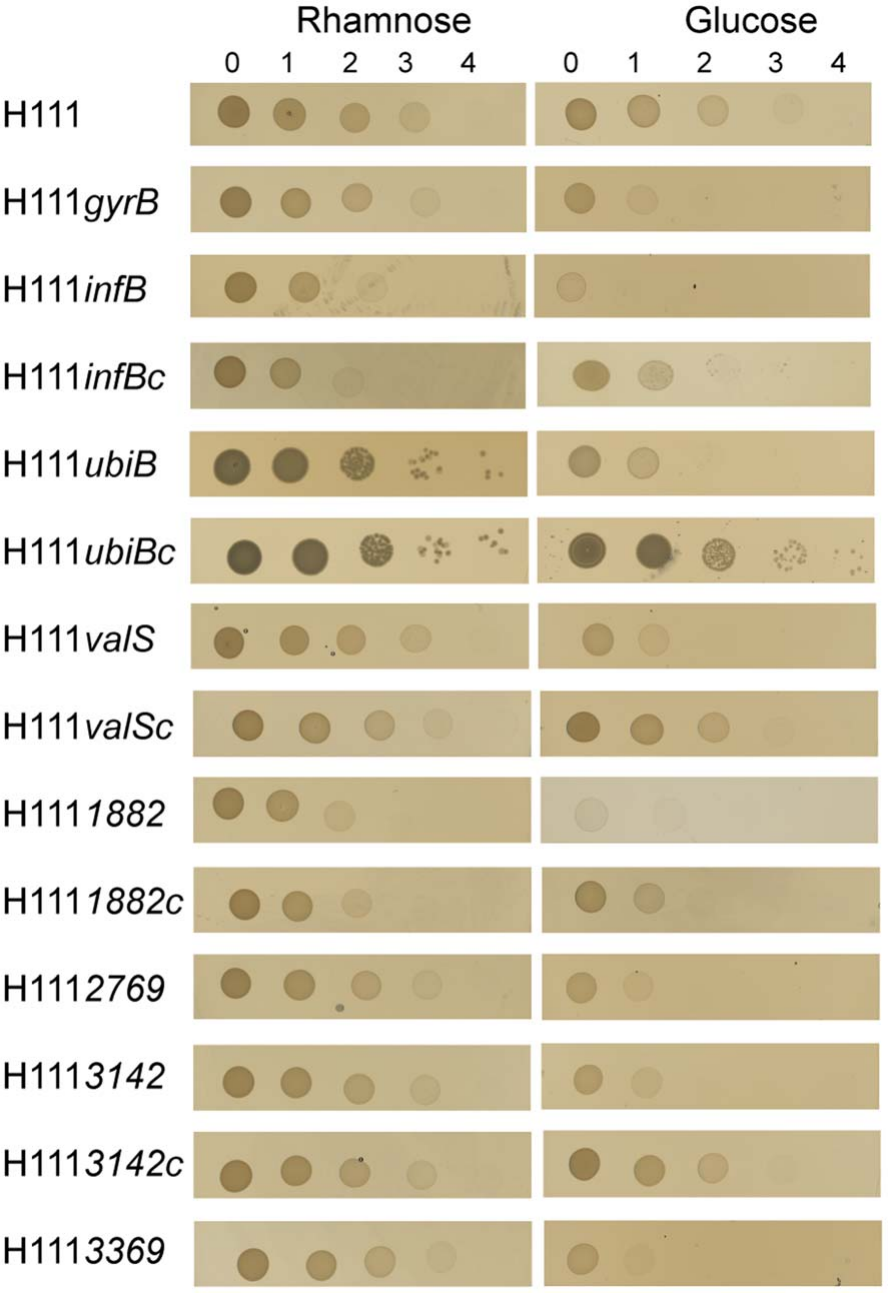
Conditional lethal phenotype of the rhamnose-dependent mutants of the *B. cenocepacia* essential genes. The constructed rhamnose-inducible mutants H111*infB*, H111*gyrB*, H111*uniB*, H111*valS*, H111*BCAL1882*, H111*BCAL2769*, H111*BCAL3142* and H111*BCAL3369* grew on LB plates supplemented with rhamnose but not with glucose as expected for mutants with essential genes under the control of rhamnose promoter. Complementation of mutants H111*infB*c, H111*ubiB*c, H111*valS*c, H111*1882*c and H111*3142*c *in trans* has restored their ability to grow on glucose. Undiluted and 10-fold diluted cultures of mutants (0, 1) usually grew visibly on plates supplemented with either glucose or rhamnose prior to depletion of the existing protein; however, at 100, 1000 and 10000- fold dilutions (2, 3, 4) mutants were unable to grow on plates supplemented with glucose enough to be seen by eye.

**Figure 3:**
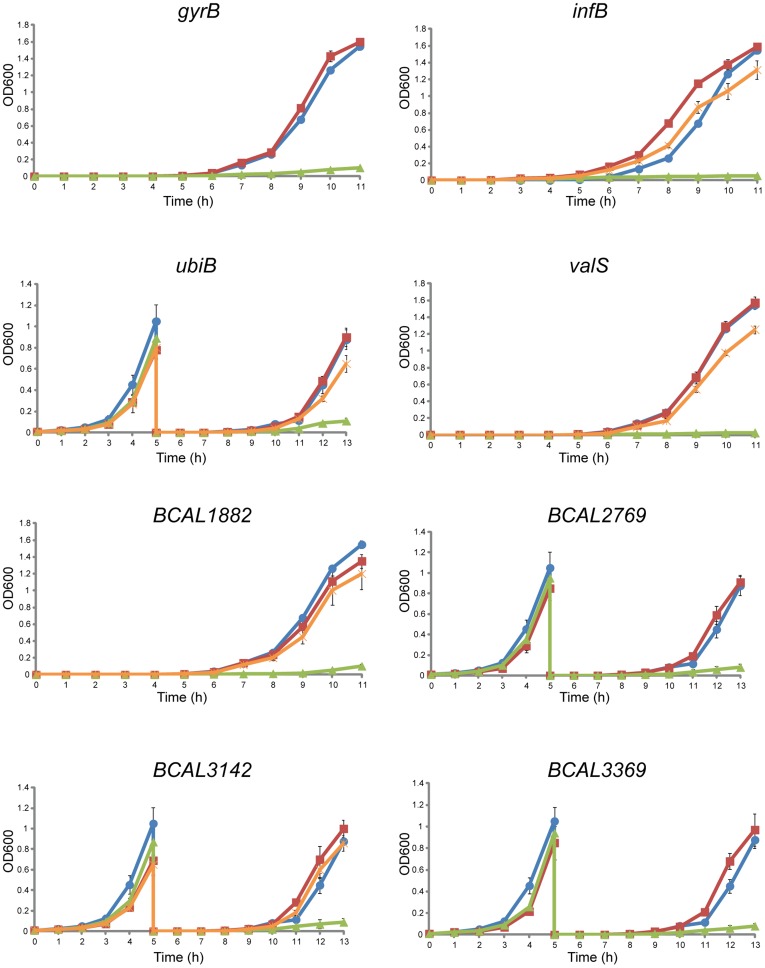
Investigated genes are essential for growth and viability of *B. cenocepacia*. Growth curves of the wild type H111 (circles), and rhamnose-inducible mutants: H111*infB*, H111*gyrB*, H111*uniB*, H111*valS*, H111*BCAL1882*, H111*BCAL2769*, H111*BCAL3142* and H111*BCAL3369* in the presence of rhamnose (squares) or glucose (triangles). Complementation of mutants H111*infB*c, H111*ubiB*c, H111*valS*c, H111*1882*c and H111*3142*c *in trans* has restored their ability to grow in the presence of glucose (stars). Values are the mean and standard deviation of a representative experiment with triplicate values.

**Figure 4:**
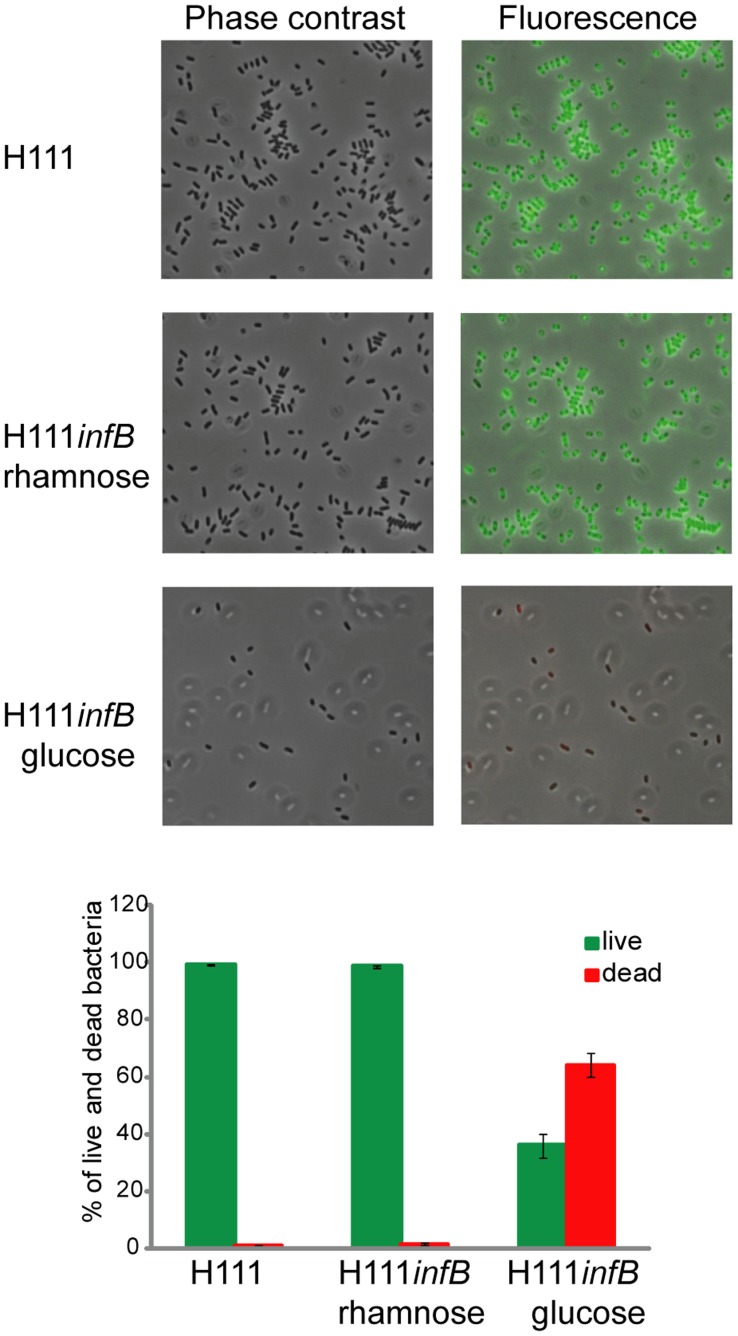
Microscopy and live-dead staining. Live-dead staining of the wild type strain H111 and of the rhamnose-inducible mutant H111*infB* grown in the presence of rhamnose or glucose. Green fluorescence indicates viable cells, while dead bacteria appear fluorescent red. The figure depicts the reduced ability of H111*infB* to survive in the medium with glucose.

The *infB* gene encodes the translation initiation factor IF-2 [45]. The constructed conditional mutant H111*infB* grew in the presence of rhamnose but not in the presence of glucose both on agar plates (Figure 2) and in liquid medium (Figure 3), as expected for a mutant with an essential gene under the control of a rhamnose-inducible promoter. To further determine whether *infB* is essential for viability, bacteria were stained with the *Bac*Light Live/Dead bacterial viability stain and examined by fluorescence microscopy. The survival rates of H111*infB* grown under permissive conditions were similar to that of the wild type (Figure 4). In contrast, the viability of the same strain grown under non-permissive conditions was greatly reduced. These results show that *infB* is not only essential for growth but also for the survival of *B. cenocepacia*. The complemented mutant grew in medium with either glucose or rhamnose, both on agar plates and in liquid medium (Figures 2 and 3), thus confirming that the observed growth impairment of the conditional mutant was caused by the mutation of *infB* and not by a polar effect on transcription of downstream genes.

The other three homologues of well-known essential genes chosen were *gyrB, ubiB*, and *valS* encoding DNA gyrase subunit [46], [47], putative ubiquinone biosynthesis protein [48], and valyl-tRNA synthetase [49], respectively. Constructed conditional rhamnose-dependent mutants H111*gyrB*, H111*ubiB*, and H111*valS* grew in the presence of rhamnose but were unable to grow in the presence of glucose on agar plates (Figure 2) or in liquid medium (Figure 3). *gyrB* is the last gene of an operon (Figure S2). Complementation of *ubiB* and *valS in trans* showed that the observed phenotypes were truly a result of the inactivation of *ubiB* and *valS* (Figures 2 and 3).

The identified core genome of *Burkholderiales* harbors also a number of completely uncharacterized hypothetical genes without assigned function, which are homologous to essential open reading frames from other organisms. To show that these uncharacterized genes are indeed crucial for viability of *B. cenocepacia*, two of them, namely *BCAL3142*, and *BCAL3369* (Figure S2) were selected for a more detailed analysis. The conditional mutants H111*BCAL3142* and H111*BCAL3369* grew well in the presence of rhamnose but were unable to grow in the presence of glucose (Figures 2 and 3), indicating that *BCAL3142* and *BCAL3369* are essential for growth of *B. cenocepacia*. *BCAL3369* is the last gene of an operon (Figure S2). Consequently the introduced rhamnose promoter regulates only expression of *BCAL3369* and thus the growth defect of H111*BCAL3369* on glucose-containing medium is caused by the inactivation of this gene. Complementation of H111*BCAL3142 in trans* showed that the observed phenotype was caused by the mutation of *BCAL3142*, and not by affecting transcription of the downstream genes (Figures 2 and 3).

These experiments suggest that a large majority of the 454 core genes of the *Burkholderiales* that are homologous to essential genes stored in the database of essential genes (DEG) are also indispensable for viability of *B. cenocepacia.*

### The Core Genome of *Burkholderia* Harbors Novel Essential Genes

Besides the 454 homologues of essential genes from other species (Table S3), the core genome of *Burkholderiales* identified also harbors 195 genes without clear orthologs in the DEG database. 111 of these genes do show weak homology to DEG genes, while the essentiality of 84 open reading frames has not been demonstrated previously (Table S4). Several of these genes yet not associated with essentiality in any studied organism are completely unknown hypotheticals with no assigned function. To investigate whether this set of 84 genes is also indispensable for cell viability, two of the uncharacterized singleton genes, namely *BCAL1882* and *BCAL2769*, were chosen randomly for further analysis. *BCAL1882* and *BCAL2769* were placed under the control of the rhamnose-inducible promoter, generating conditional mutants H111*BCAL1882* and H111*BCAL2769*. The effect of the mutation on the viability of *B. cenocepacia* was examined by growing the strains in medium with either rhamnose or glucose. The two conditional mutants grew well in the presence of rhamnose but were unable to grow in the presence of glucose on agar plates (Figure 2) as well as in liquid medium (Figure 3). Given that *BCAL2769* is not part of an operon (Figure S2), we concluded that this gene is essential for growth of *B. cenocepacia*. As *BCAL1882* is part of a large operon (Figure S2) comprising 17 genes with 11 genes located downstream of *BCAL1882*, the mutant was complemented. This experiment revealed that the observed phenotype was indeed caused by the mutation of *BCAL1882* (Figures 2 and 3).

Open reading frames *BCAL1882* and *BCAL2769*, together with the six genes described in the previous section, *infB, gyrB, ubiB, valS, BCAL3142* and *BCAL3369*, have been chosen randomly from the identified 649 core genes. Experimental proof of their indispensability in *B. cenocepacia*, together with the fact that the vast majority (454) genes have homologues in the database of essential genes leads to the conclusion that the core genome identified in our study is composed mostly or exclusively of genes which are essential. Although examples of highly conserved non-essential genes have been described in literature (e.g. *recA*), these genes may provide the investigated organism with a fitness advantage under certain specific environmental or laboratory conditions. The core genes identified in our study are likely indispensable for survival of *B. cenocepacia* in its natural environment, but not all of them are necessarily essential under certain laboratory conditions. Perhaps the most interesting aspect of the identification of the core genome is that it harbours a number of genes not associated with essentiality in any previously studied organism. We believe that these novel essential genes are of particular interest, as they may be exploited as potential targets for the development of novel antimicrobials and their further study may help to better understand essential cellular functions.

### Chromosome 1 of *Burkholderia* is Crucial for the Storage of Essential Genes

The genomes of all representatives of the genus *Burkholderia* investigated so far consist of more than one chromosome, and with annotated genome sizes ranging from 6 to 9 Mb belong among the largest genomes observed among Gram-negative bacteria [30]. The genomes of potential bio-terrorism agents, *B. mallei* and *B. pseudomallei* contain two chromosomes, with the larger chromosome 1 (4.1 Mb and 3.5 Mb, respectively) encoding mostly genes involved in primary metabolism and growth and the smaller chromosome 2 (3.2 Mb and 2.3 Mb, respectively) harboring genes involved in adaptation to different niches [28]. The complete genome of *B. cenocepacia* J2315, which was used as a reference strain in our genome analysis, contains three circular chromosomes of 3.9, 3.2 and 0.9 Mb and a plasmid of 92.7 Kb encoding 7261 predicted open reading frames in total. Interestingly, examination of the location of the core genes identified in this study revealed that the vast majority of them are located in the largest chromosome of *B. cenocepacia* J2315 (636/649) (Figure 1). Out of the remaining 13 genes, only two are singletons (genes with no homologues within the genome) and are located on chromosome 2 (Figure 1). This is in full agreement with a previous study that demonstrated that chromosomes 2 and 3 of *B. cenocepacia* J2315 harbor mostly genes encoding accessory functions [29].

### Conclusions

Soon after the genome sequences of the first two sequenced bacteria *Haemophilus influen*zae and *Mycoplasma genitalium* have become available, comparative genomics was employed to predict essential genes [50]. The rationale of this *in silico* approach was that genes that are conserved between two evolutionarily distant organisms are likely to be essential. In this first study 250 candidate essential genes were identified. However, as more genome sequences became available in the following years, the number of conserved genes decreased continually [9], [10], [50]–[52]. Most recent work suggested that the universal core of essential genes consists of less than 50 genes [53]. This number of genes, however, is doubtless too low to code for all the essential functions of a living cell [4]. This apparent discrepancy indicates that either the homology of essential genes was below the threshold value used for the identification of orthologous genes or that some essential functions are dependent on phylogenetically unrelated sets of proteins, as it is the case with isoenzymes. Given that gene sequence homologies are lower between phylogenetically unrelated organisms, the evolutionary distance between analysed genomes can have a significant impact on the outcome of the comparative genome analyses [4]. To avoid these problems, we decided to determine the core genome of a group of phylogenetically closely related organisms, namely of the order *Burkholderia*. We also reasoned that this approach may identify essential genes that are only present within this order. Non-orthologous gene displacement has the potential to constrain the coverage/false-negative rate of such analysis. However, this phenomenon can only generate false negatives, no false positives. In addition, while non-orthologous gene displacement is well described and well supported, it is a rather rare event and will affect only few genes (especially when limiting the analysis to a single order).

The genome comparison studies have been recently performed for several bacterial species, including *Bordetella* [54], [55], *Bifidobacterium* [56], *Escherichia* and *Salmonella* [57], and *Streptococcus* [58]. The analysis of the core genome of *Bifidobacterium*, comparing nine sequenced *Bifidobacterium* genomes, provided novel insight how these bacteria adapt to the conditions in the human gastrointestinal tract [56]. Comparison of the whole genome sequences of *Escherichia* and *Salmonella* revealed a remarkable sequence similarity of genes horizontally acquired by *Escherichia* and *Salmonella* suggesting that these were derived from a common source, a supraspecies pangenome of horizontally shared genes [57].

Of the 649 *Burkholderiales* core genes identified in our study, many (454) were previously shown to be essential in at least one other organism. The essentiality of 6 of these genes (*infB, gyrB, ubiB, valS, BCAL3142* and *BCAL3369*) for *B. cenocepacia* was confirmed within this study. Intriguingly, we also identified 84 genes, which so far have not been found to be essential in another organism. In fact, the presence of many of these genes appears to be restricted to the order *Burkholderiales* or organisms in the phylogenetic proximity of this order. We provide experimental evidence that two of these genes, uncharacterized and randomly chosen, *BCAL1882* and *BCAL2769* are essential for *B. cenocepacia*. *BCAL1882* encodes an entirely unknown protein. The only available information concerning *BCAL2769* is its putative nucleotide binding property and cytoplasmic localization, suggesting that it might be involved in basic biological processes related to information storage and processing. Elucidation of exact biological functions of *BCAL1882* and *BCAL2769* with the help of the constructed conditional mutants is currently on the way in our laboratory. The vast majority of the *B. cenocepacia* essential genes identified are located on the chromosome 1, thus underlying the importance of chromosome 1 for the encoding of the “house-keeping” essential functions. Our data indicate that several of the essential genes are conserved within an evolutionary lineage and are not present or not detectable in phylogenetically unrelated bacteria. This suggests that some essential functions may have independently evolved; in other words it appears that evolutionary different solutions to the same problem exist. For example, it is obvious that an intact cell membrane is essential for every living cell, yet the structures of bacterial cell walls are strikingly different and it is likely that their biosynthesis will depend on enzymes which share little if any homology. Intriguingly, many of the identified *B. cenocepacia* essential genes that are conserved in members of the *Burkholderiales* are of unknown function but are predicted to be either outer membrane proteins or possibly involved in cell wall biosynthesis. In summary, our results suggest that the core genomes of phylogenetically related organisms may allow a more reliable prediction of essential genes than those previously determined for very distantly related organisms.

## Materials and Methods

### Bacterial Strains, Plasmids, and Growth Conditions

Bacterial strains and plasmids used in this study are listed in Table 1. In most cases *E. coli* and *B. cenocepacia* grew in Luria-Bertani or SOB medium. In some cases *B cenocepacia* was grown in PIA (Pseudomonas isolation medium containing 2% glycerol) or in the semi-defined medium outlined in by Ortega et al [43]. When required these media were supplemented with 0.5% rhamnose, 0.5% glucose, or trimethoprim (50 µg/ml or 100 µg/ml). Liquid cultures grew on a rotatory shaker at 220 rpm and 37°C.

**Table 1:** Bacterial strains and plasmids used in this study.

Strain or plasmid	Characteristics	Reference
*B. cenocepacia*		
H111	Wild type	Lab. collection
H111*engA*	*engA* mutant of H111	This study
H111*gyrB*	*gyrB* mutant of H111	This study
H111*infB*	*infB* mutant of H111	This study
H111*ubiB*	*ubiB* mutant of H111	This study
H111*valS*	*valS* mutant of H111	This study
H111*1882*	*BCAL1882* mutant of H111	This study
H111*2430*	*BCAM2430* mutant of H111	This study
H111*2769*	*BCAL2769* mutant of H111	This study
H111*3142*	*BCAL3142* mutant of H111	This study
H111*3369*	*BCAL3369* mutant of H111	This study
H111*engAc*	H111*engA* complemented with pBBRMCS2*engA*	This study
H111*infB*c	H111*infB* complemented with pBBRMCS2*infBw*	This study
H111*ubiB*c	H111*ubiB* complemented with pBBRMCS2*ubiBw*	This study
H111*valS*c	H111*valS* complemented with pBBRMCS2*valSw*	This study
H111*1882*c	H111*1882* complemented with pBBRMCS2*1882w*	This study
H111*3142*c	H111*3142* complemented with pBBRMCS2*3142w*	This study
*E.coli*		
CC118	λ pir	Lab. collection
TOP10		Lab. collection
Plasmids		
pSC200	P_rhaB_ (rhamnose-inducible), Tp^r^	[43]
pSC200*engA*	pSC200 carrying fragment of *engA*	This study
pSC200*gyrB*	pSC200 carrying fragment of *gyrB*	This study
pSC200*infB*	pSC200 carrying fragment of *infB*	This study
pSC200*ubiB*	pSC200 carrying fragment of *ubiB*	This study
pSC200*valS*	pSC200 carrying fragment of *valS*	This study
pSC200*1882*	pSC200 carrying fragment of *1882*	This study
pSC200*2430*	pSC200 carrying fragment of *2430*	This study
pSC200*2769*	pSC200 carrying fragment of *2769*	This study
pSC200*3142*	pSC200 carrying fragment of *3142*	This study
pSC200*3369*	pSC200 carrying fragment of *3369*	This study
pBBRMCS2*engAw*	pBBRMCS2 carrying *engA*	This study
pBBRMCS2*infBw*	pBBRMCS2 carrying *infB*	This study
pBBRMCS2*ubiBw*	pBBRMCS2 carrying *ubiB*	This study
pBBRMCS2*valSw*	pBBRMCS2 carrying *valS*	This study
pBBRMCS2*1882w*	pBBRMCS2 carrying *1882*	This study
pBBRMCS2*3142w*	pBBRMCS2 carrying *3142*	This study

### Recombinant DNA Methodology

Restriction endonucleases and T4 DNA ligase were obtained from Roche and Invitrogen respectively. DNA extractions, plasmid isolations and gel purifications were performed with the DNeasy tissue kit, Qiaprep Spin Miniprep kit and Qiaquick gel extraction kit (Qiagen) respectively, according to manufacturer’s instructions. Oligonucleotide primers were synthesized by Eurofins MWG. Recombinant DNA techniques were performed as described by Sambrook [59]. Standard PCR amplifications were performed in 10 µl reaction mixtures using *Taq* DNA polymerase (Qiagen), HotStar *Taq* polymerase (Qiagen) or ProofStart DNA polymerase (Fermentas).

### Generation of Rhamnose-dependent Conditional Mutants of *B. cenocepacia* Essential Genes

Approximately 300 bp fragments of target genes starting at the start codon were amplified, digested and ligated into pSC200 digested with the appropriate restriction endonucleases. Recombinant plasmids were transformed into chemically competent *E. coli* CC118 λ pir and successful transformants were recovered on a media supplemented with 50 µg/ml trimethoprim and 0.5% glucose. Subsequently the recombinant plasmids were transferred into *B. cenocepacia* by triparental mating where by homologous recombination the native promoters of target genes were replaced with the rhamnose-inducible promoter introduced into the chromosome by the plasmid. The conditional mutants were selected on PIA medium supplemented with 0.5% rhamnose and 100 µg/ml trimethoprim. Constructed plasmids and *Burkholderia* conditional mutants are listed in Table 1.

### Complementation of Conditional Mutants

Plasmids pBBRMCS2*infBw*, pBBRMCS2*ubiBw*, pBBRMCS2*valSw*, pBBRMCS2*1882w* and pBBRMCS2*3142w* were generated by cloning the whole gene sequences of ORFs: *infB, ubiB, valS, BCA1882* and *BCAL3142* into the broad host-range shuttle vector pBBRMCS2. The recombinant plasmids used for complementation of conditional mutants *in trans* were introduced into *E. coli* TOP10 chemically competent cells by transformation and subsequently into the H111*infB,* H111*ubiB,* H111*valS,* H111*1882* and H111*3142* conditional mutants by triparental mating. The mutants H111*infB*c, H111*ubiB*c, H111*valS*c, H111*1882*c, and H111*3142*c complemented *in trans* were selected on PIA medium supplemented with.5% rhamnose, 100 µg/ml trimethoprim and 50 µg/ml kanamycin.

### Bacterial Growth and Viability Assay

Bacterial strains grew overnight using a rotatory shaker at 220 rpm and 37°C in liquid LB supplemented with 0.2% rhamnose. 2 ml aliquots of the overnight culture were centrifuged, the pellet was washed several times with PBS. Bacterial cells were adjusted to an optical density OD_600_ of 1.0 and serially diluted up to 10^−4^. To compare growth of conditional mutants on solid media, 10 µl drops from each dilutions were transferred on LB media supplemented with either 0.5% glucose or 0.5% rhamnose and incubated for 9–11 h at 37°C. To investigate the growth defect of essential genes’ conditional mutants in liquid media, the 300 µl of the 10^−2^ dilution were inoculated into 30 ml of liquid LB media (final OD_600_ = 0.0001 ) supplemented with either 0.5% glucose or 0.5% rhamnose and incubated for 9–11 h on a rotatory shaker at 220 rpm and 37°C. In some instances it was necessary to deplete the product of the target gene which accumulated in bacterial cells from overnight cultivation in rhamnose. To do this, bacteria were inoculated into liquid LB media supplemented with either 0.5% glucose or 0.5% rhamnose to an OD_600_ = 0.01 and incubated for 5 hours to an OD_600_ = 1.0. Subsequently, bacterial cells were washed several times with PBS and inoculated into fresh liquid media supplemented with either 0.5% glucose or 0.5% rhamnose to an OD OD_600_ = 0.0005 and incubated on a rotatory shaker at 220 rpm and 37°C for additional 8–10 hours. The viability of *B. cenocepacia* strains in liquid culture was determined by using a *Bac*Light Live/Dead bacterial viability staining kit (Molecular Probes Inc., Leiden, Netherlands). Two stocks of stains (green-fluorescent nucleic acid stain SYTO9 and red-fluorescent nucleic acid stain propidium iodide) were each diluted to a concentration of 3 µl/ml in a medium. These stains differ in their ability to penetrate healthy bacterial cells. SYTO9 labels all bacteria in a population, while propidium iodide penetrates only non-viable cells with damaged membranes, causing a reduction of the SYTO 9 stain fluorescence when both dyes are present [60], [61]. Live SYTO9- stained bacteria and dead propidium- stained bacteria after 11 h of growth were observed in a fluorescence microscope and the means and standard deviations were calculated from three representative images.

### Sequences and Databases

The sequences of the previously identified sets of essential genes from other bacterial species, namely *Pseudomonas aeruginosa* strain PA14 [14], *Bacillus subtilis* [8] and *Escherichia coli* strain MG1655 [38] were obtained from the Database of Essential Genes (DEG 6.5) (http://tubic.tju.edu.cn/deg/) [39], [40], [62]. The annotated genome of the *Burkholderia cenocepacia* strain J2315 was downloaded from the website of the *Burkholderia* sequencing project of the Sanger Institute, UK (http://www.sanger.ac.uk/resources/downloads/bacteria/burkholderia-cenocepacia.html) [29]. The genomes sequences of all the other *Burkholderia* species were obtained from the website of the *Burkholderia* Genome Database (http://www.burkholderia.com) [63].

### *In Silico* Identification of the Core Genome of *Burkholderiaceae*

We downloaded the complete proteomes of 51 members of the order *Burkholderiales* from the STRING 9 Database (Table S1). After an all-against-all BLAST of these proteomes (e− value cutoff 10e−5), the OrthoMCL implementation of similarity matrices [64] and Markov Clustering was used to establish the orthologous groups (50% match). A set of 610 orthologous groups (containing 649 genes in *B. cenocepacia* J2315) was detected to be present in all 51 proteomes (Table S2).

### Identification of Homologues of Essential Genes and Novel Essential Genes in *B. cenocepacia*

The sequences of previously identified essential genes were concatenated into three sets, each representing group of essential genes of different bacterium, *P. aeruginosa*, *B. subtilis* and *E. coli*. The concatenated sets of previously identified essential genes of *P. aeruginosa*, *B. subtilis* and *E. coli* were annotated according to information stored in the Database of Essential Genes (DEG 5.4) [39], [40] and visualized with Artemis [65], a sequence viewer and annotation tool that allows visualization of sequence features as well as the results of analyses within the context of the sequence, and its six-frame translation. Sequences of sets of previously identified sets of essential genes of *P. aeruginosa*, *B. subtilis* and *E. coli* and of genes of the core genome of *Burholderiaceae* were compared using the Artemis comparison tool (ACT) (http://www.webact.org/WebACT/home) [65], [66] to identify regions of homology by pairwise comparison using the TBLASTX algorithm (minimum cut-off 150 for *P. aeruginosa* and *B subtilis* and 200 for *E. coli*). Homologues of genes from the DEG database on the B. *cenocepacia* J2315 genome were detected with a reciprocal BLAST analysis (minimum bitscore 100) across the genomes as implemented in the OrthoMCL software [64]. The application of this criterion revealed 195 genes of the core genome of *Burholderiaceae* that seemed to have no counterpart in the DEG database. Thus we considered them as genes, which had potentially not yet been described as essential. To reduce the number of potentially overlooked known essentials in this list, we removed genes that had distant homologues in the DEG database (the minimum blast score for removal was 50). Additional 8 genes, which after manual investigation we considered to be already known essentials were removed as well from the final list. This processing yielded the final set of 84 core genes, which have not yet been found to be essential in other organisms. Enrichment of DEG homologues in our core genome was detected using a Chi- squared test (chromosome 1) and a Fisher’s exact test (chromosome 2).

## Supporting Information

Figure S1 **Generation of conditional mutants.** Conditional mutants used to investigate essentiality of selected *B. cenocepacia* genes were generated by replacement of the candidate essential genes native promoters for the rhamnose-inducible promoter. Short fragments (300 bp) of investigated genes were cloned into pSC200 downstream of the plasmid-borne rhamnose promoter. Promoters were exchanged by transfer of recombinant plasmids into *B. cenocepacia* by triparental mating and homologous recombination.
(TIF)

Figure S2 **Genetic organization of essential genes.** Figure shows the genetic organization of the chosen *B. cenocepacia* essential genes and their flanking regions. The black arrows indicate the locations of the inserted rhamnose-inducible promoter.
(TIF)

Figure S3 **Control growth curves.** Growth curves of the *B. cenocepacia* wild type H111 and rhamnose-inducible mutants in two genes which are not part of the core genome identified: H111*2430* and H111*engA* in the presence of rhamnose (squares) or glucose (triangles). The growth of *B. cenocepacia* H111 strain in permissive and non-permissive conditions was unaltered, thus showing that the presence of rhamnose or glucose in the medium does not have any effect on the growth of *B. cenocepacia* H111. Conditional mutant H111*2430* grew in the presence of either rhamnose or glucose. Conditional mutant H111*engA* grew in rhamnose but was unable to grow in glucose similarly to mutants in essential genes. However, complementation of mutant H111*engAc in trans* did not restore its ability to grow in glucose (stars), thus showing that the growth deficiency of H111*engA* was not a result of essentiality of *engA* but rather of polar effects on downstream genes. Values are the mean and standard deviation of a representative experiment with triplicate values.
(TIF)

Table S1 **Genomes of** ***Burkholderiales.***
(DOC)

Table S2 **Core genome of the order** ***Burkholderiales*** **.**
(DOC)

Table S3 ***Burkholderiales*** **core genome DEG homologs.**
(DOC)

Table S4 **Novel essential genes.**
(DOC)
