# Supplementary figures and images for: High Confidence Prediction of Essential Genes in Burkholderia Cenocepacia
Source: PLoS One. 2012 Jun 29;7(6):e40064. doi: 10.1371/journal.pone.0040064 (PMC3386938; doi:10.1371/journal.pone.0040064)

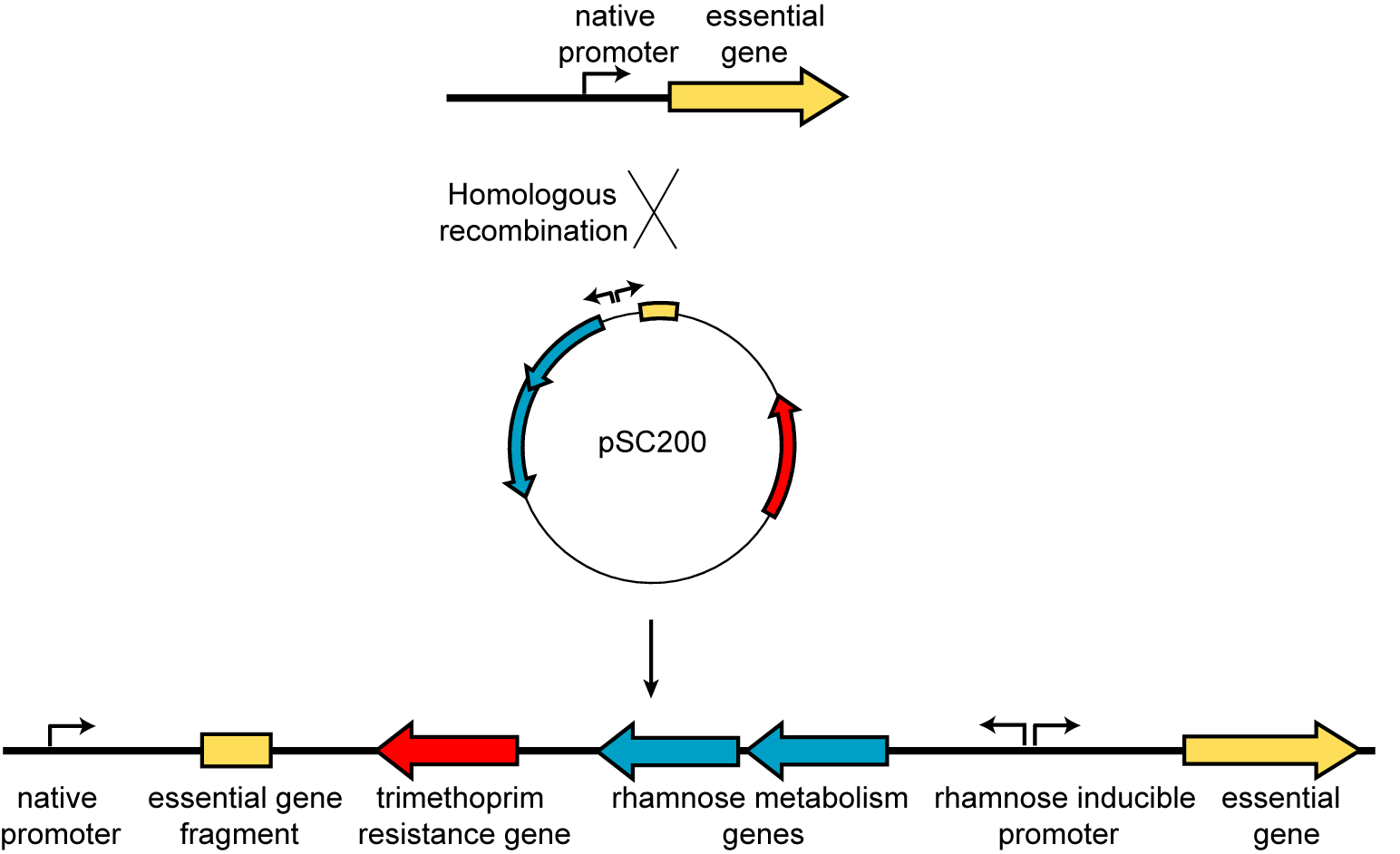

Supplement: Figure S1 — Generation of conditional mutants. Conditional mutants used to investigate essentiality of selected B. cenocepacia genes were generated by replacement of the candidate essential genes native promoters for the rhamnose-inducible promoter. Short fragments (300 bp) of investigated genes were cloned into pSC200 downstream of the plasmid-borne rhamnose promoter. Promoters were exchanged by transfer of recombinant plasmids into B. cenocepacia by triparental mating and homologous recombination. (TIF) [file pone.0040064.s001.tif]

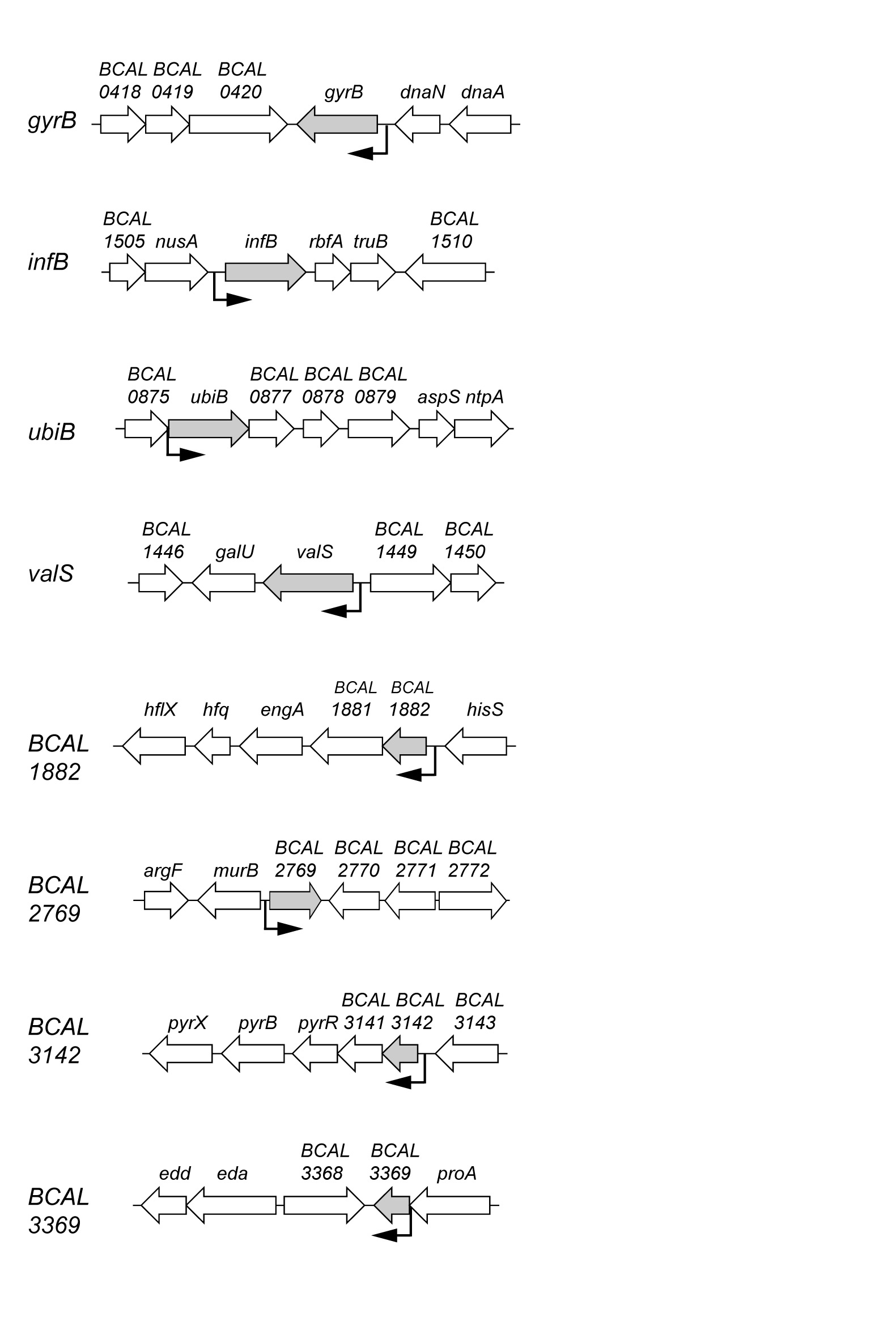

Supplement: Figure S2 — Genetic organization of essential genes. Figure shows the genetic organization of the chosen B. cenocepacia essential genes and their flanking regions. The black arrows indicate the locations of the inserted rhamnose-inducible promoter. (TIF) [file pone.0040064.s002.tif]

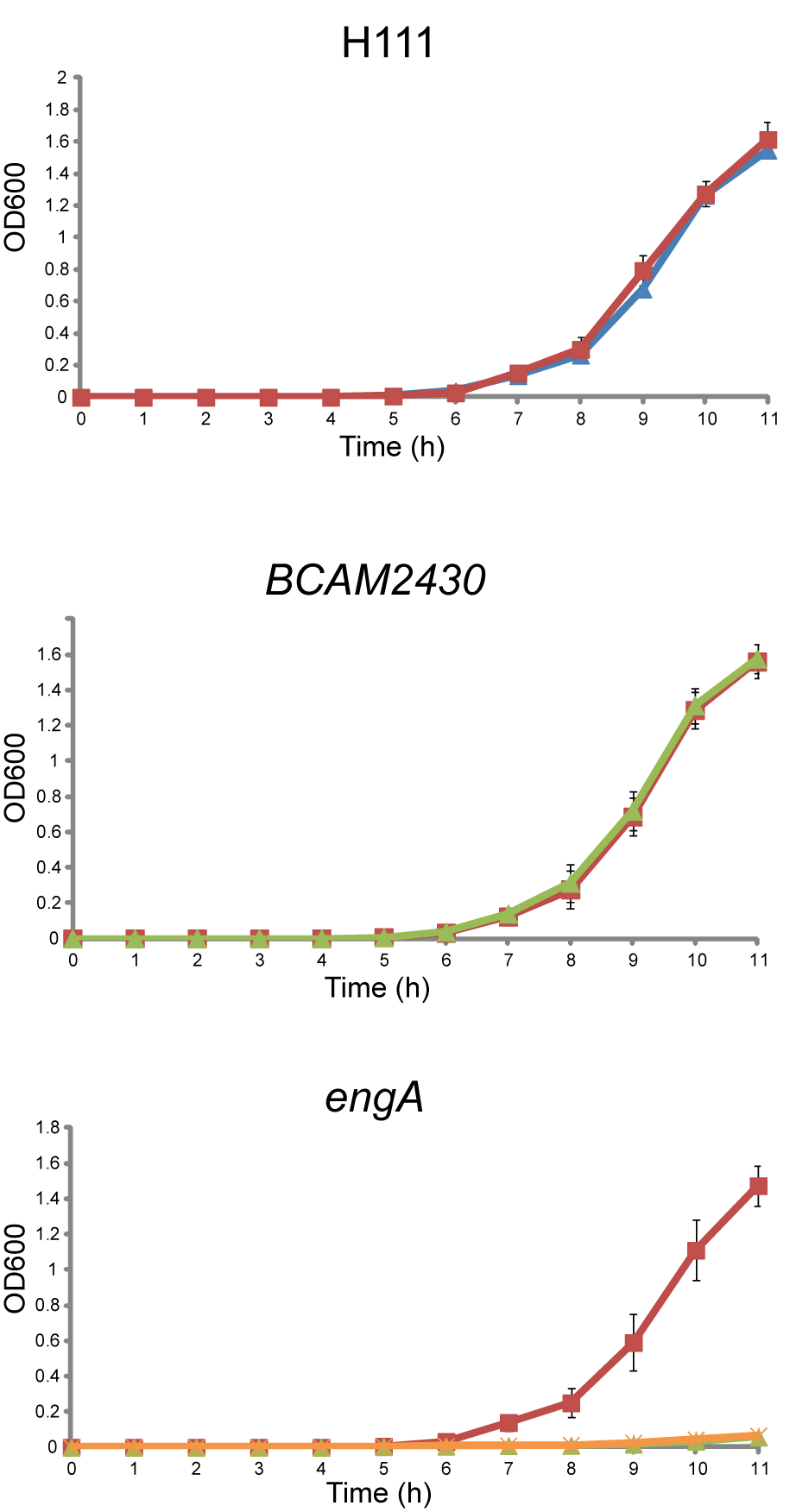

Supplement: Figure S3 — Control growth curves. Growth curves of the B. cenocepacia wild type H111 and rhamnose-inducible mutants in two genes which are not part of the core genome identified: H1112430 and H111engA in the presence of rhamnose (squares) or glucose (triangles). The growth of B. cenocepacia H111 strain in permissive and non-permissive conditions was unaltered, thus showing that the presence of rhamnose or glucose in the medium does not have any effect on the growth of B. cenocepacia H111. Conditional mutant H1112430 grew in the presence of either rhamnose or glucose. Conditional mutant H111engA grew in rhamnose but was unable to grow in glucose similarly to mutants in essential genes. However, complementation of mutant H111engAc in trans did not restore its ability to grow in glucose (stars), thus showing that the growth deficiency of H111engA was not a result of essentiality of engA but rather of polar effects on downstream genes. Values are the mean and standard deviation of a representative experiment with triplicate values. (TIF) [file pone.0040064.s003.tif]
