# Supplementary material for: High Confidence Prediction of Essential Genes in Burkholderia Cenocepacia
Source: PLoS One. 2012 Jun 29;7(6):e40064. doi: 10.1371/journal.pone.0040064 (PMC3386938; doi:10.1371/journal.pone.0040064)
Supplement: Table S1 — Genomes of Burkholderiales. (DOC) [file pone.0040064.s004.doc]

**Table S1. Genomes of *Burkholderiales*.**

| *Acidovorax citrulli* |
| --- |
| *Acidovorax sp. JS42* |
| *Bordetella avium* |
| *Bordetella bronchiseptica* |
| *Bordetella parapertussis* |
| *Bordetella petrii* |
| *Bordetella pertussis* |
| *Burkholderia ambifaria AMMD* |
| *Burkholderia ambifaria MC40* |
| *Burkholderia cenocepacia J2315* |
| *Burkholderia cenocepacia 1054* |
| *Burkholderia cenocepacia 2424* |
| *Burkholderia cenocepacia MC03* |
| *Burkholderia glumae* |
| *Burkholderia mallei 10229* |
| *Burkholderia mallei 10247* |
| *Burkholderia mallei 3344* |
| *Burkholderia mallei SAVP1* |
| *Burkholderia multivorans 17616* |
| *Burkholderia phymatum STM815* |
| *Burkholderia phytofirmans PsJN* |
| *Burkholderia pseudomallei 1106a* |
| *Burkholderia pseudomallei 1710b* |
| *Burkholderia pseudomallei 668* |
| *Burkholderia pseudomallei K96243* |
| *Burkholderia pseudomallei MSHR346* |
| *Burkholderia sp. 383* |
| *Burkholderia thailandensis* |
| *Burkholderia vietnamiensis* |
| *Burkholderia xenovorans* |
| *Comamonas testosteroni* |
| *Cupriavidus taiwanensis* |
| *Diaphorobacter sp. TPSY* |
| *Delftia acidovorans* |
| *Herminiimonas arsenicoxydans* |
| *Janthinobacterium sp. Marseille* |
| *Leptothrix cholodnii* |
| *Methylibium petroleiphilum* |
| *Polaromonas naphthalenivorans* |
| *Polaromonas sp. JS666* |
| *Polynucleobacter necessarius QLW* |
| *Polynucleobacter necessarius STIR1* |
| *Ralstonia eutropha H16* |
| *Ralstonia eutropha JMP134* |
| *Ralstonia metallidurans* |
| *Ralstonia pickettii 12D* |
| *Ralstonia pickettii 12J* |
| *Ralstonia solanacearum* |
| *Rhodoferax ferrireducens* |
| *Variovorax paradoxus* |
| *Verminephrobacter eiseniae* |

Table shows 51 members of the order *Burkholderiales* whose genomes have been compared in this study.
