# Supplementary material for: High Confidence Prediction of Essential Genes in Burkholderia Cenocepacia
Source: PLoS One. 2012 Jun 29;7(6):e40064. doi: 10.1371/journal.pone.0040064 (PMC3386938; doi:10.1371/journal.pone.0040064)
Supplement: Table S2 — Core genome of the order Burkholderiales . (DOC) [file pone.0040064.s005.doc]

**Table S2. Core genome of the order *Burkholderiales*.**

| **Locus tag** | **Gene name** | **Product** |
| --- | --- | --- |
| BCAL0006 |  | putative protein lysine methyltransferase protein |
| BCAL0024 | *gidA* | tRNA urid 5-carboxymethylaminomet mod enz GidA |
| BCAL0025 | *gidB* | 16S rRNA methyltransferase GidB |
| BCAL0026 | *parA* | chromosome partitioning protein ParA |
| BCAL0027 | *parB* | chromosome partitioning protein ParB |
| BCAL0030 | *atpB* | F0F1 ATP synthase subunit A |
| BCAL0031 | *atpE* | F0F1 ATP synthase subunit C |
| BCAL0033 | *atpH* | F0F1 ATP synthase subunit delta |
| BCAL0034 | *atpA* | F0F1 ATP synthase subunit alpha |
| BCAL0035 | *atpG* | F0F1 ATP synthase subunit gamma |
| BCAL0036 | *atpD* | F0F1 ATP synthase subunit beta |
| BCAL0037 | *atpC* | F0F1 ATP synthase subunit epsilon |
| BCAL0041 | *priA* | primosome assembly protein PriA |
| BCAL0115 | *rpsU1* | 30S ribosomal protein S21 |
| BCAL0145 | *ahcY* | S-adenosyl-L-homocysteine hydrolase |
| BCAL0147 | *metF* | 5,10-methylenetetrahydrofolate reductase |
| BCAL0160 |  | putative methylase |
| BCAL0163 |  | putative phospholipid-binding lipoprotein |
| BCAL0219 | *tufA1* | elongation factor Tu |
| BCAL0220 | *secE* | preprotein translocase subunit SecE |
| BCAL0221 | *nusG* | transcription antitermination protein NusG |
| BCAL0222 | *rplK* | 50S ribosomal protein L11 |
| BCAL0223 | *rplA* | 50S ribosomal protein L1 |
| BCAL0224 | *rplJ* | 50S ribosomal protein L10 |
| BCAL0225 | *rplL* | 50S ribosomal protein L7/L12 |
| BCAL0227 | *rpoC* | DNA-directed RNA polymerase subunit beta |
| BCAL0229 | *rpsL* | 30S ribosomal protein S12) |
| BCAL0230 | *rpsG* | 30S ribosomal protein S7 |
| BCAL0231 | *fusA* | elongation factor G |
| BCAL0232 | *tuf* | elongation factor Tu |
| BCAL0233 | *rpsJ* | 30S ribosomal protein S10 |
| BCAL0234 | *rplC* | 50S ribosomal protein L3 |
| BCAL0235a | *rplD* | 50S ribosomal protein L4 |
| BCAL0236 | *rplW* | 50S ribosomal protein L23 |
| BCAL0237 | *rplB* | 50S ribosomal protein L2 |
| BCAL0238 | *rpsS* | 30S ribosomal protein S19 |
| BCAL0239a | *rplV* | 50S ribosomal protein L22 |
| BCAL0240 | *rpsC* | 30S ribosomal protein S3 |
| BCAL0241 | *rplP* | 50S ribosomal protein L16 |
| BCAL0242 | *rpmC* | 50S ribosomal protein L29 |
| BCAL0243 | *rpsQ* | 30S ribosomal protein S17 |
| BCAL0244 | *rplN* | 50S ribosomal protein L14 |
| BCAL0246 | *rplE* | 50S ribosomal protein L5 |
| BCAL0247 | *rpsN* | 30S ribosomal protein S14 |
| BCAL0248 | *rpsH* | 30S ribosomal protein S8 |
| BCAL0249 | *rplF* | 50S ribosomal protein L6 |
| BCAL0250 | *rplR* | 50S ribosomal protein L18 |
| BCAL0251 | *rpsE* | 30S ribosomal protein S5 |
| BCAL0252 | *rpmD* | 50S ribosomal protein L30 |
| BCAL0253 | *rplO* | 50S ribosomal protein L15 |
| BCAL0254 | *secY* | preprotein translocase subunit SecY |
| BCAL0257 | *rpsM* | 30S ribosomal protein S13 |
| BCAL0258 | *rpsK* | 30S ribosomal protein S11 |
| BCAL0259 | *rpsD* | 30S ribosomal protein S4 |
| BCAL0260 | *rpoA* | DNA-directed RNA polymerase subunit alpha |
| BCAL0261 | *rplQ* | 50S ribosomal protein L17 |
| BCAL0264 | *hemB* | delta-aminolevulinic acid dehydratase |
| BCAL0266 |  | putative cytochrome c4 |
| BCAL0273 | *cyaY* | frataxin-like protein |
| BCAL0274 | *mrcA* | penicillin-binding protein 1A |
| BCAL0280 | *aroB* | 3-dehydroquinate synthase |
| BCAL0299 | *thiG* | thiazole synthase |
| BCAL0301 |  | ABC transporter ATP-binding protein |
| BCAL0302 |  | hypothetical protein |
| BCAL0303 |  | hypothetical protein |
| BCAL0309 |  | BolA-like protein |
| BCAL0310 | *murA* | UDP-N-acetylglucosamine 1-carboxyvinyltransf |
| BCAL0312 | *hisD* | histidinol dehydrogenase |
| BCAL0314 | *hisB* | imidazoleglycerol-phosphate dehydr |
| BCAL0316 | *hisH* | imidazole glycerol phosphate synthase sub HisH |
| BCAL0317 | *hisA* | 1-(5-phosphor)-5-[(5-phosphorib imid-4-carbox isom |
| BCAL0318 | *hisF* | imidazole glycerol phosphate synthase sub HisF |
| BCAL0319 | *hisI* | phosphoribosyl-AMP cyclohydrolase |
| BCAL0320 | *hisE* | phosphoribosyl-ATP pyrophosphatase |
| BCAL0323 | *tatA* | twin arginine translocase protein A |
| BCAL0324 | *tatB* | sec-independent translocase |
| BCAL0325 | *tatC* | Sec-independent protein translocase protein TatC |
| BCAL0328 | *petA* | ubiquinol-cytochrome c reductase iron-sulfur subunit |
| BCAL0329 | *petB* | cytochrome b |
| BCAL0330 | *petC* | cytochrome c1 precursor |
| BCAL0331 |  | putative stringent starvation protein A |
| BCAL0332 |  | ClpXP protease specificity-enhancing factor |
| BCAL0373 | *hemK* | protein methyltransferase HemK |
| BCAL0374 | *prfA* | peptide chain release factor 1 |
| BCAL0375 | *hemA* | glutamyl-tRNA reductase |
| BCAL0387 | *engD* | GTP-dependent nucleic acid-binding prot EngD |
| BCAL0388 |  | ubiquinone biosynthesis hydroxylase family prot |
| BCAL0389 | *dsbC* | thiol:disulfide interchange protein DsbC |
| BCAL0390 |  | metallo peptidase, family M61 |
| BCAL0396 | *trpC* | indole-3-glycerol-phosphate synthase |
| BCAL0397 | *trpD* | anthranilate phosphoribosyltransferase |
| BCAL0398 | *trpG* | anthranilate synthase component II |
| BCAL0399 | *trpE* | anthranilate synthase component I |
| BCAL0401 |  | ribulose-phosphate 3-epimerase |
| BCAL0402 | *apaG* | ApaG |
| BCAL0403 |  | put outer membr-bound lytic murein transglycosyl |
| BCAL0409 | *paaF* | enoyl-CoA hydratase |
| BCAL0421 | *gyrB* | DNA gyrase subunit B |
| BCAL0422 | *dnaN* | DNA polymerase III subunit beta |
| BCAL0423 | *dnaA* | chromosomal replication initiation protein |
| BCAL0425 |  | hypothetical protein |
| BCAL0426 |  | put inner membr prot translocase component YidC |
| BCAL0428 | *trmE* | tRNA modification GTPase TrmE |
| BCAL0460 |  | D-isomer specific 2-hydroxyacid dehydrogenase |
| BCAL0462 |  | DNA topoisomerase III |
| BCAL0464 |  | SMF family protein |
| BCAL0465 | *def* | peptide deformylase |
| BCAL0466 | *fmt* | methionyl-tRNA formyltransferase |
| BCAL0470 |  | hypothetical protein |
| BCAL0471 |  | two-component regulatory system |
| BCAL0472 |  | two-component regulatory system |
| BCAL0473 |  | putative putative transcriptional regulator |
| BCAL0478 | *rodA* | rod shape-determining protein |
| BCAL0479 | *mrdA* | penicillin-binding protein |
| BCAL0481 | *mreC* | rod shape-determining protein MreC |
| BCAL0482 | *mreB* | rod shape-determining protein MreB |
| BCAL0483 | *gatC* | aspartyl/glutamyl-tRNA amidotransferase sub C |
| BCAL0484 | *gatA* | aspartyl/glutamyl-tRNA amidotransferase sub A |
| BCAL0485 | *gatB* | aspartyl/glutamyl-tRNA amidotransferase sub B |
| BCAL0487 |  | endonuclease/exonuclease/phosphatase family prot |
| BCAL0492 | *metW* | methionine biosynthesis protein MetW |
| BCAL0493 | *metX* | homoserine O-acetyltransferase |
| BCAL0496 | *argB* | acetylglutamate kinase |
| BCAL0502 |  | DksA/TraR C4-type zinc finger family protein |
| BCAL0503 |  | putative cobalamin synthesis protein |
| BCAL0505 | *xerC* | site-specific tyrosine recombinase XerC |
| BCAL0506 |  | hypothetical protein |
| BCAL0508 |  | lipid A biosynthesis lauroyl acyltransferase |
| BCAL0509 | *metK* | S-adenosylmethionine synthetase) |
| BCAL0537 |  | endonuclease/exonuclease/phosphatase fam prot |
| BCAL0554 |  | 5-formyltetrahydrofolate cyclo-ligase family protein |
| BCAL0555 |  | putative transglycosylase |
| BCAL0558 | *cca* | Multif tRNA nucleot tr /2'3'-cyc phosph/2'nucl/phos |
| BCAL0611 | *glmS1* | glucosamine--fructose-6-phosphate aminotransferase |
| BCAL0612 | *glmU* | bifunctional glmU protein |
| BCAL0613 |  | hypothetical protein |
| BCAL0614 |  | hypothetical protein |
| BCAL0677 | *dsbA* | thiol:disulfide interchange protein |
| BCAL0679 | *argS* | arginyl-tRNA synthetase |
| BCAL0709 | *lipB* | lipoyltransferase |
| BCAL0710 | *lipA* | lipoyl synthase |
| BCAL0729 | *glnB1* | nitrogen regulatory protein P-II 1 |
| BCAL0734 |  | sugar transport PTS system IIa component |
| BCAL0735 | *ptsH* | phosphocarrier protein HPr |
| BCAL0736 |  | put phosphoenolpyruvate-protein phosphotransf |
| BCAL0738 |  | C-terminal processing protease-3 |
| BCAL0739 | *gpmA* | phosphoglycerate mutase |
| BCAL0741 | *grxC* | glutaredoxin 3 |
| BCAL0742 | *secB* | preprotein translocase subunit SecB |
| BCAL0743 | *gpsA* | NAD(P)H-dep glycerol-3-phosphate dehydrog |
| BCAL0745 |  | SpoU rRNA methylase family protein |
| BCAL0758 |  | putative cytochrome oxidase assembly prot |
| BCAL0759 |  | protoheme IX farnesyltransferase |
| BCAL0760 |  | probable lipoprotein |
| BCAL0787 | *sigM* | RNA polymerase factor sigma-32 |
| BCAL0793 |  | putative cell division protein |
| BCAL0794 |  | hypothetical protein |
| BCAL0795 | *coaD* | phosphopantetheine adenylyltransferase |
| BCAL0796 |  | 4Fe-4S ferredoxin |
| BCAL0798 | *pth* | peptidyl-tRNA hydrolase |
| BCAL0799 | *ctc* | 50S ribosomal protein L25/general stress prot Ctc |
| BCAL0800 | *prs* | ribose-phosphate pyrophosphokinase |
| BCAL0804 |  | hypothetical protein |
| BCAL0805 | *mutM* | formamidopyrimidine-DNA glycosylase |
| BCAL0806 | *mutY* | putative A/G-specific adenine glycosylase |
| BCAL0808 |  | hypothetical protein |
| BCAL0809 |  | HPr kinase/phosphorylase |
| BCAL0814 |  | ABC transporter ATP-binding protein |
| BCAL0816 |  | hypothetical protein |
| BCAL0818 |  | putative arabinose 5-phosphate isomerase |
| BCAL0825 | *uvrA* | excinuclease ABC subunit A |
| BCAL0827 | *ssb* | single-stranded DNA-binding protein |
| BCAL0869 | *ilvA* | threonine dehydratase |
| BCAL0870 |  | putative oxidoreductase |
| BCAL0873 | *ubiE* | ubiquinone/menaquinone biosynth methyltransf |
| BCAL0876 | *ubiB* | putative ubiquinone biosynthesis protein UbiB |
| BCAL0880 | *aspS* | aspartyl-tRNA synthetase |
| BCAL0892 |  | putative nucleotidyl transferase |
| BCAL0893 |  | phosphotransferase enzyme family protein |
| BCAL0894 |  | hypothetical protein |
| BCAL0895 |  | putative peptidyl-prolyl cis-trans isomerase |
| BCAL0897 | *ksgA* | dimethyladenosine transferase |
| BCAL0901 |  | putative acyltransferase |
| BCAL0903 | *glyS* | glycyl-tRNA synthetase subunit beta |
| BCAL0904 | *glyQ* | glycyl-tRNA synthetase subunit alpha |
| BCAL0907 |  | putative cation transporter efflux protein |
| BCAL0909 |  | unkown domain/put metalloprotease fusion prot |
| BCAL0911 | *miaB* | putative tRNA thiotransferase protein MiaB |
| BCAL0953 | *recA* | recombinase A |
| BCAL0956 | *sucC* | succinyl-CoA synthetase subunit beta |
| BCAL0957 | *sucD* | succinyl-CoA synthetase subunit alpha |
| BCAL0958 |  | hypothetical protein |
| BCAL0963 |  | metallo peptidase, subfamily M48B |
| BCAL0968 |  | hypothetical protein |
| BCAL0971 |  | 4Fe-4S ferredoxin |
| BCAL0980 | *mobA* | molybdopterin-guanine dinucle biosynthesis prot A |
| BCAL0982 | *rne2* | putative ribonuclease E |
| BCAL0987 |  | putative tetrapyrrole methylase |
| BCAL0988 |  | Maf-like protein |
| BCAL0990 | *rpmF* | 50S ribosomal protein L32 |
| BCAL0991 | *plsX2* | put glycerol-3-phosphate acyltransferase PlsX |
| BCAL0992 | *fabH2* | 3-oxoacyl-(acyl carrier protein) synthase III |
| BCAL0993 | *fabD2* | malonyl CoA-acyl carrier protein transacylase 2 |
| BCAL0994 | *fabG* | 3-ketoacyl-(acyl-carrier-protein) reductase |
| BCAL0995 | *acpP* | acyl carrier protein |
| BCAL0996 | *fabF2* | 3-oxoacyl-(acyl carrier protein) synthase II |
| BCAL1001 | *mucD2* | serine protease MucD 2 |
| BCAL1003 | *lepA2* | GTP-binding protein LepA |
| BCAL1004 | *lepB2* | signal peptidase I 2 (leader peptidase Lep 2) |
| BCAL1005 | *rnc2* | ribonuclease III |
| BCAL1006 | *era* | GTP-binding protein Era |
| BCAL1010 | *nagZ2* | beta-hexosaminidase |
| BCAL1012 | *efp2* | elongation factor P |
| BCAL1013 |  | hypothetical protein |
| BCAL1019 | *pgsA2* | put CDP-diacylglyc--glyc-3-phosph 3-phosphatidyltr 2 |
| BCAL1033 |  | hypothetical protein |
| BCAL1254 | *dnaQ* | DNA polymerase III subunit epsilon |
| BCAL1255 | *rnhA* | ribonuclease HI |
| BCAL1257 |  | putative gultathione hydrolase |
| BCAL1262 | *carB* | carbamoyl phosphate synthase large subunit |
| BCAL1263 | *greA* | transcription elongation factor GreA |
| BCAL1265 |  | hypothetical protein |
| BCAL1266 |  | put rib RNA large sub methyltransf |
| BCAL1267 | *ftsH* | FtsH endopeptidase |
| BCAL1269 |  | putative phosphoglucomutase |
| BCAL1270 | *pstS* | phosphate transport system |
| BCAL1274 |  | phosphate transport system-related protein |
| BCAL1275 | *phoB* | phosphate regulon two-component regulatory syst |
| BCAL1276 |  | two-component reg syst, sensor kinase protein |
| BCAL1321 | *glmS3* | glucosam--fruct-6-phosp aminotr [isomerizing] 3 |
| BCAL1413 | *glnS* | glutaminyl-tRNA synthetase |
| BCAL1416 | *alaS* | alanyl-tRNA synthetase |
| BCAL1441 |  | SirA-like protein |
| BCAL1448 | *valS* | valyl-tRNA synthetase |
| BCAL1449 |  | putative helicase |
| BCAL1460 |  | putative FAD-binding reductase |
| BCAL1463 |  | putative tRNA processing exoribonuclease |
| BCAL1467 | *aroC* | chorismate synthase |
| BCAL1468 |  | putative electron transport protein |
| BCAL1473 | *scoB* | succinyl-CoA:3-ketoacid-coenzyme A transf sub B |
| BCAL1478 |  | putative hydrolase |
| BCAL1481 | *thrS* | threonyl-tRNA synthetase |
| BCAL1484 | *rplT* | 50S ribosomal protein L20 |
| BCAL1485 | *pheS* | phenylalanyl-tRNA synthetase alpha chain |
| BCAL1486 | *pheT* | phenylalanyl-tRNA synthetase subunit beta |
| BCAL1487 | *ihfA* | integration host factor subunit alpha |
| BCAL1488 |  | MerR family regulatory protein |
| BCAL1503 |  | putative transcriptional regulator protein |
| BCAL1504 |  | RNA pseudouridylate synthase family protein |
| BCAL1505 |  | hypothetical protein |
| BCAL1506 | *nusA* | transcription elongation factor NusA |
| BCAL1507 | *infB* | translation initiation factor IF-2 |
| BCAL1508 | *rbfA* | ribosome-binding factor A |
| BCAL1509 | *truB* | tRNA pseudouridine synthase B |
| BCAL1515 | *sucA* | 2-oxoglutarate dehydrogenase E1 component |
| BCAL1516 | *sucB* | dihydrolipoamide succinyltransferase |
| BCAL1517 | *odhL* | dihydrolipoamide dehydrogenase |
| BCAL1518 |  | AFG1-like ATPase |
| BCAL1541 |  | putative acyl-CoA synthetase |
| BCAL1554 |  | putative tRNA/rRNA methyltransferase |
| BCAL1556 | *rpiA* | ribose-5-phosphate isomerase A |
| BCAL1611 | *pyrD* | dihydroorotate dehydrogenase 2 |
| BCAL1612 |  | arginyl-tRNA-protein transferase |
| BCAL1614 |  | NUDIX hydrolase |
| BCAL1625 |  | tRNA-dihydrouridine synthase A |
| BCAL1651 | *lexA* | LexA repressor |
| BCAL1779 |  | ABC transporter ATP-binding protein |
| BCAL1825 |  | hypothetical protein |
| BCAL1852 |  | putative phosphoserine phosphatase |
| BCAL1860 | *pbhF* | put polyhydroxyalkan (PHA) synt reg prot |
| BCAL1863 | *pbhC* | poly-beta-hydroxybutyrate polymerase |
| BCAL1864 |  | hypothetical protein |
| BCAL1865 |  | ribosomal large subunit pseudouridine synthase D |
| BCAL1873 | *purA* | adenylosuccinate synthetase |
| BCAL1874 | *hisZ* | ATP phosphoribosyltransferase regulatory subunit |
| BCAL1877 | *hflK* | protein HflK |
| BCAL1881 |  | putative lipoprotein |
| BCAL1882 |  | hypothetical protein |
| BCAL1883 | *hisS* | histidyl-tRNA synthetase |
| BCAL1884 | *ispG* | 4-hydroxy-3-methylbut-2-en-1-yl diphosph synth |
| BCAL1886 |  | radical SAM superfamily protein |
| BCAL1887 | *ndk* | nucleoside diphosphate kinase |
| BCAL1888 |  | hypothetical protein |
| BCAL1889 | *rumA* | 23S rRNA 5-methyluridine methyltransferase |
| BCAL1893 |  | family M23 peptidase |
| BCAL1894 |  | putative L-isoaspartate O-methyltransferase |
| BCAL1897 | *recR* | recombination protein RecR |
| BCAL1899 | *dnaX* | DNA polymerase III subunits gamma and tau |
| BCAL1900 | *trxA* | thioredoxin |
| BCAL1901 | *rho* | transcription termination factor Rho |
| BCAL1905 | *rpmE2* | 50S ribosomal protein L31 type B |
| BCAL1906 |  | hypothetical protein |
| BCAL1919 | *clpB* | ClpB heat-shock protein |
| BCAL1922 | *moaE* | molybdopterin converting factor subunit 2 |
| BCAL1924 | *moeA3* | molybdopterin biosynthesis protein MoeA 3 |
| BCAL1925 | *thrC* | threonine synthase |
| BCAL1926 | *hom* | homoserine dehydrogenase |
| BCAL1936 |  | AhpC/TSA family protein |
| BCAL1941 | *dnaB* | replicative DNA helicase |
| BCAL1942 | *rplI* | 50S ribosomal protein L9 |
| BCAL1943 | *rpsR* | 30S ribosomal protein S18 |
| BCAL1944 |  | putative primosomal replication protein |
| BCAL1945 | *rpsF* | 30S ribosomal protein S6 |
| BCAL1962 |  | putative deoxyribonuclease |
| BCAL1963 |  | DNA polymerase III subunit delta' |
| BCAL1964 | *tmk* | thymidylate kinase |
| BCAL1965 |  | putative lipoprotein |
| BCAL1966 | *ygfZ* | tRNA-modifying protein YgfZ |
| BCAL1970 |  | thioesterase superfamily protein |
| BCAL1981 |  | hypothetical protein |
| BCAL1983 |  | intracellular septation protein A |
| BCAL1985 |  | putative exported isomerase |
| BCAL1989 |  | putative carbohydrate kinase |
| BCAL1992 |  | putative acyl-CoA thioesterase precursor |
| BCAL1993 |  | putative peptidyl-prolyl cis-trans isomerase |
| BCAL1994 | *Ion* | ATP-dependent protease La |
| BCAL1995 | *clpX* | ATP-dependent protease ATP-binding subunit ClpX |
| BCAL1996 | *clpP* | ATP-dependent Clp protease proteolytic subunit |
| BCAL1997 | *tig* | trigger factor; Involved in protein export |
| BCAL2016 | *ispD* | 2-C-met-D-erythritol 4-phosphate cytidylyltr |
| BCAL2017 | *mfd* | transcription-repair coupling factor |
| BCAL2021 |  | putative penicillin-binding protein |
| BCAL2044 | *lcdA* | L,D-carboxypeptidase A |
| BCAL2061 | *guaA* | GMP synthase |
| BCAL2063 | *guaB* | inosine 5'-monophosphate dehydrogenase |
| BCAL2071 | *smpB* | SsrA-binding protein |
| BCAL2075 |  | hypothetical protein |
| BCAL2076 |  | putative RNA methylase protein |
| BCAL2077 | *rnhB* | ribonuclease HII |
| BCAL2078 | *lpxB* | lipid-A-disaccharide synthase |
| BCAL2079 | *lpxA* | UDP-N-acetylglucosamine acyltransferase |
| BCAL2081 | *lpxD* | UDP-3-O-[3-hydroxymyristoyl] glucosam N-acyltr |
| BCAL2082 |  | chaperone protein Skp precursor |
| BCAL2083 |  | Outer membrane protein assembly factor YaeT |
| BCAL2084 | *ecfE* | protease EcfE |
| BCAL2085 | *dxr* | 1-deoxy-D-xylulose 5-phosphate reductoisomerase |
| BCAL2087 | *uppS* | undecaprenyl pyrophosphate synthetase |
| BCAL2088 | *frr* | ribosome recycling factor |
| BCAL2089 | *pyrH* | uridylate kinase |
| BCAL2090 | *tsf* | elongation factor Ts |
| BCAL2091 | *rpsB* | 30S ribosomal protein S2 |
| BCAL2092 | *map* | methionine aminopeptidase |
| BCAL2094 |  | putative RNA pseudouridylate synthase |
| BCAL2096 | *ligA* | DNA ligase |
| BCAL2097 |  | hypothetical protein |
| BCAL2101 | *dapD* | 2,3,4,5-tetrahydropyrid-2,6-carbox N-succinyltr |
| BCAL2103 | *dapE* | succinyl-diaminopimelate desuccinylase |
| BCAL2104 |  | N5-glutam S-adenosyl-L-methionine-dep methyltr |
| BCAL2109 | *radA* | DNA repair protein RadA |
| BCAL2110 | *alr* | alanine racemase |
| BCAL2117 |  | putative ATP-dependent RNA helicase |
| BCAL2119 |  | universal stress protein family protein |
| BCAL2126 | *gltX* | glutamyl-tRNA synthetase |
| BCAL2129 |  | ABC transporter ATP-binding protein |
| BCAL2146 | *ask* | aspartate kinase |
| BCAL2147 |  | tRNA(Ile)-lysidine synthase |
| BCAL2148 | *accA* | acetyl-CoA carboxylase carboxyltr subunit alpha |
| BCAL2149 |  | HhH-GPD superfam base excision DNA repair prot |
| BCAL2150 | *cysS* | cysteinyl-tRNA synthetase |
| BCAL2153 | *ppiB* | peptidyl-prolyl cis-trans isomerase B oligopept |
| BCAL2154 |  | UDP-2,3-diacylglucosamine hydrolase |
| BCAL2156 |  | putative tRNA/rRNA methyltransferase protein |
| BCAL2157 | *suhB* | inositol-1-monophosphatase |
| BCAL2166 |  | putative lipoprotein |
| BCAL2167 | *dapA* | dihydrodipicolinate synthase |
| BCAL2172 |  | putative phosphoesterase |
| BCAL2175 |  | hypothetical protein |
| BCAL2178 | *ftsB* | cell division protein FtsB |
| BCAL2179 | *eno* | phosphopyruvate hydratase |
| BCAL2180 |  | 2-dehydro-3-deoxyphosphooctonate aldolase |
| BCAL2181 | *pyrG* | CTP synthetase |
| BCAL2184 |  | putative TatD related DNase |
| BCAL2186 |  | pute lipoprot releasing system transmembr prot |
| BCAL2190 | *lysS* | lysyl-tRNA synthetase |
| BCAL2194 | *hscA* | chaperone protein HscA |
| BCAL2195 | *hscB* | co-chaperone HscB |
| BCAL2196 |  | HesB family protein |
| BCAL2197 |  | scaffold protein |
| BCAL2198 | *iscS* | cysteine desulfurase |
| BCAL2199 |  | putative transcriptional regulator protein |
| BCAL2204 |  | IclR family regulatory protein |
| BCAL2206 | *phaP* | phasin-like protein |
| BCAL2207 |  | putative dihydrolipoamide dehydrogenase |
| BCAL2208 | *pdhB* | dihydrolipoamide acetyltransferase |
| BCAL2209 | *aceE* | pyruvate dehydrogenase subunit E1 |
| BCAL2212 | *folD* | FolD bifunctional protein |
| BCAL2213 |  | oligopeptidase A |
| BCAL2220 |  | endonuclease/exonuclease/phosphatase fam prot |
| BCAL2222 | *glnG* | nitrogen regulation protein NR(I) |
| BCAL2224 | *glnA* | glutamine synthetase |
| BCAL2227 |  | hypothetical protein |
| BCAL2232 |  | hypothetical protein |
| BCAL2328 |  | hypothetical protein |
| BCAL2331 | *nuoN* | NADH dehydrogenase subunit N |
| BCAL2332 | *nuoM* | NADH dehydrogenase subunit M |
| BCAL2333 | *nuoL* | NADH dehydrogenase subunit L |
| BCAL2334 | *nuoK* | NADH dehydrogenase subunit K |
| BCAL2335 | *nuoJ* | NADH dehydrogenase subunit J |
| BCAL2336 | *nuoI* | NADH dehydrogenase subunit I |
| BCAL2337 | *nuoH* | NADH dehydrogenase subunit H |
| BCAL2338 | *nuoG* | NADH dehydrogenase subunit G |
| BCAL2339 | *nuoF* | NADH dehydrogenase I chain F |
| BCAL2340 | *nuoE* | NADH dehydrogenase subunit E |
| BCAL2341 | *nuoD* | NADH dehydrogenase subunit D |
| BCAL2342 | *nuoC* | NADH dehydrogenase subunit C |
| BCAL2343 | *nuoB* | NADH dehydrogenase subunit B |
| BCAL2344 | *nuoA* | NADH dehydrogenase subunit A |
| BCAL2346 | *tpiA* | triosephosphate isomerase |
| BCAL2348 | *pnp* | polynucleotide phosphorylase/polyadenylase |
| BCAL2349 | *rpsO* | 30S ribosomal protein S15 |
| BCAL2355 |  | putative phosphatidyltransferase |
| BCAL2357 | *ilvC* | ketol-acid reductoisomerase |
| BCAL2358 | *ilvH* | acetolactate synthase 3 regulatory subunit |
| BCAL2359 | *ilvI* | acetolactate synthase 3 catalytic subunit |
| BCAL2389 | *purD* | phosphoribosylamine--glycine ligase |
| BCAL2390 | *hemF* | coproporphyrinogen III oxidase |
| BCAL2392 |  | hypothetical protein |
| BCAL2393 |  | rRNA large subunit methyltransferase |
| BCAL2394 |  | Maf-like protein |
| BCAL2408 | *msbA* | lipid A export ATP-binding/permease protein MsbA |
| BCAL2409 | *dnaE* | DNA polymerase III subunit alpha |
| BCAL2412 | *rhlE2* | putative ATP-dependent RNA helicase 2 |
| BCAL2417 |  | putative DNA translocase |
| BCAL2420 |  | putative depolymerase/histone-like protein |
| BCAL2424 |  | ferredoxin |
| BCAL2425 | *nth* | endonuclease III |
| BCAL2430 |  | putative ATPases |
| BCAL2433 | *talB* | transaldolase B |
| BCAL2454 | *parC* | DNA topoisomerase IV subunit A |
| BCAL2455 | *parE* | DNA topoisomerase IV subunit B |
| BCAL2456 |  | ABC transporter ATP-binding protein |
| BCAL2618 |  | hypothetical protein |
| BCAL2622 | *ppa* | inorganic pyrophosphatase |
| BCAL2638 | *argH* | argininosuccinate lyase |
| BCAL2641 |  | putative ornithine decarboxylase |
| BCAL2642 | *dcd* | deoxycytidine triphosphate deaminase |
| BCAL2644 |  | putative ATP-binding protein |
| BCAL2646 | *metG* | methionyl-tRNA synthetase |
| BCAL2650 |  | put chromosome condensation and segregation prot |
| BCAL2651 | *panC* | pantoate--beta-alanine ligase |
| BCAL2675 |  | DNA polymerase III subunit chi |
| BCAL2676 | *pepA* | leucyl aminopeptidase |
| BCAL2677 |  | putative permease protein |
| BCAL2678 |  | putative permease protein |
| BCAL2686 | *cysB* | transcriptional regulator CysB-like protein |
| BCAL2700 |  | CDP-6-deoxy-delta-3,4-glucoseen reductase |
| BCAL2705 |  | ABC transporter ATP-binding protein |
| BCAL2710 | *ispH* | 4-hydroxy-3-methylbut-2-enyl diphosphate reductase |
| BCAL2714 | *rpmB* | 50S ribosomal protein L28 |
| BCAL2715 | *rpmG* | 50S ribosomal protein L33 |
| BCAL2719 |  | putative transmembrane fatty acid desaturase |
| BCAL2721 |  | NOL1/NOP2/Sun family protein |
| BCAL2724 | *ileS* | isoleucyl-tRNA synthetase |
| BCAL2725 | *lspA* | lipoprotein signal peptidase |
| BCAL2726 | *dfp* | bif phosphopantothcyst decarbox/phosphopant synt |
| BCAL2731 | *clpS* | ATP-dependent Clp protease adaptor protein ClpS |
| BCAL2739 | *fusA* | elongation factor G |
| BCAL2758 | *xseA* | exodeoxyribonuclease VII large subunit |
| BCAL2759 |  | tetraacyldisaccharide 4'-kinase |
| BCAL2761 | *kdsB* | 3-deoxy-manno-octulosonate cytidylyltransferase |
| BCAL2762 | *adk* | adenylate kinase |
| BCAL2764 |  | MviN-like protein |
| BCAL2767 | *argF* | ornithine carbamoyltransferase |
| BCAL2768 | *murB* | UDP-N-acetylenolpyruvoylglucosamine reduct |
| BCAL2769 |  | putative nucleotide-binding protein |
| BCAL2770 |  | putative glycerol-3-phosphate acyltransf PlsY |
| BCAL2773 | *xerD* | site-specific tyrosine recombinase XerD |
| BCAL2782 | *pdxH* | pyridoxamine 5'-phosphate oxidase |
| BCAL2788 |  | flavin reductase family prot |
| BCAL2836 | *purK* | phosphoribosylaminoimidazole carbox ATPase sub |
| BCAL2837 | *purE* | phosphoribosylaminoimidazole carbox catalyt sub |
| BCAL2838 | *purC* | phosphoribosylaminoimidaz-succinocarboxam synt |
| BCAL2839 | *cbbA* | fructose-1,6-bisphosphate aldolase |
| BCAL2841 | *pgk* | phosphoglycerate kinase |
| BCAL2853 | *pgsA1* | CDP-diacylglyc--glyc-3-phosph 3-phosphatidyltr 1 |
| BCAL2857 |  | hypothetical protein |
| BCAL2858 | *efp1* | elongation factor P |
| BCAL2860 | *nagZ1* | beta-hexosaminidase |
| BCAL2864 | *era* | GTP-binding protein Era |
| BCAL2865 | *rnc1* | ribonuclease III |
| BCAL2866 | *lepB1* | signal peptidase I 1 (leader peptidase Lep 1) |
| BCAL2867 | *lepA1* | GTP-binding protein LepA |
| BCAL2869 | *mucD1* | serine protease MucD 1 |
| BCAL2874 | *fabF1* | 3-oxoacyl-(acyl carrier protein) synthase II |
| BCAL2875 | *acpP* | acyl carrier protein |
| BCAL2876 | *fabG* | 3-ketoacyl-(acyl-carrier-protein) reductase |
| BCAL2877 | *fabD1* | malonyl CoA-acyl carrier protein transacylase 1 |
| BCAL2878 | *fabH1* | 3-oxoacyl-(acyl carrier protein) synthase III |
| BCAL2879 | *plsX1* | putative glycerol-3-phosphate acyltransferase PlsX |
| BCAL2880 | *rpmF* | 50S ribosomal protein L32 |
| BCAL2882 |  | Maf-like protein |
| BCAL2883 |  | putative tetrapyrrole methylase |
| BCAL2888 | *rne1* | ribonuclease E 1 |
| BCAL2890 | *mobA* | molybdopterin-guanine dinucleot biosynth prot A |
| BCAL2899 |  | 4Fe-4S ferredoxin |
| BCAL2912 | *thyA* | thymidylate synthase |
| BCAL2915 | *dfrA* | dihydrofolate reductase |
| BCAL2916 |  | microcin-processing peptidase |
| BCAL2920 |  | metallo peptidase, subfamily M48A |
| BCAL2921 |  | ribosome-associated GTPase |
| BCAL2925 | *rplS* | 50S ribosomal protein L19 |
| BCAL2926 | *trmD* | tRNA (guanine-N(1)-)-methyltransferase |
| BCAL2927 | *rimM* | 16S rRNA-processing protein RimM |
| BCAL2934 | *etfA* | electron transfer flavoprotein alpha-subunit |
| BCAL2935 | *etfB* | electron transfer flavoprotein beta-subunit |
| BCAL2941 |  | putative exported transglycosylase |
| BCAL2947 |  | tetratricopeptide repeat protein |
| BCAL2949 | *ihfB* | integration host factor subunit beta |
| BCAL2950 | *rpsA* | 30S ribosomal protein S1 |
| BCAL2952 | *aroA* | 3-phosphoshikimate 1-carboxyvinyltransferase |
| BCAL2954 | *pheA* | P-protein |
| BCAL2955 | *serC* | phosphoserine aminotransferase |
| BCAL2957 | *gyrA* | DNA gyrase subunit A |
| BCAL2958 | *ompA* | putative OmpA family protein |
| BCAL2959 | *ubiG* | 3-demethylubiquinone-9 3-methyltransferase |
| BCAL2960 |  | putative phosphatase |
| BCAL2993 | *pepN* | aminopeptidase N |
| BCAL2994 | *fbp* | fructose-1,6-bisphosphatase |
| BCAL3010 | *spoT* | guanosine-3',5'-bis(diphosp) 3'-pyrophosphohydr |
| BCAL3011 | *rpoZ* | DNA-directed RNA polymerase subunit omega |
| BCAL3012 | *gmk* | guanylate kinase |
| BCAL3016 |  | coproporphyrinogen III oxidase |
| BCAL3030 | *serS* | seryl-tRNA synthetase |
| BCAL3032 | *rarA* | recombination factor protein RarA |
| BCAL3033 | *lolA* | outer-membrane lipoprotein carrier protein |
| BCAL3034 | *ftsK* | DNA translocase FtsK |
| BCAL3035 | *trxB* | thioredoxin reductase |
| BCAL3049 | *hemL* | glutamate-1-semialdehyde 2,1-aminomutase |
| BCAL3050 | *ribD* | multifunctional riboflavin biosynthetic protein |
| BCAL3051 | *ribE* | riboflavin synthase subunit alpha |
| BCAL3053 | *ribB* | bif3,4-dihydr-2-but4-pho synt/GTP cyclohydr II- prot |
| BCAL3054 | *ribH* | 6,7-dimethyl-8-ribityllumazine synthase |
| BCAL3057 |  | putative lipoprotein |
| BCAL3090 | *trmB* | tRNA (guanine-N(7)-)-methyltransferase |
| BCAL3110 | *waaA* | 3-deoxy-D-manno-octulosonic-acid transferase |
| BCAL3132 | *rmlD* | dTDP-4-keto-L-rhamnose reductase |
| BCAL3135 | *rmlB* | dTDP-D-glucose 4,6-dehydratase |
| BCAL3136 | *apaH* | diadenosine tetraphosphatase |
| BCAL3141 |  | Holliday junction resolvase-like protein |
| BCAL3142 |  | hypothetical protein |
| BCAL3146 | *groEL* | chaperonin GroEL |
| BCAL3147 | *groES* | co-chaperonin GroES |
| BCAL3196 | *nrdR* | transcriptional regulator NrdR |
| BCAL3197 | *glyA* | serine hydroxymethyltransferase |
| BCAL3200 | *tolQ* | putative TolQ transport transmembrane protein |
| BCAL3201 | *tolR* | putative TolR-related protein |
| BCAL3203 | *tolB* | translocation protein TolB |
| BCAL3204 |  | putative OmpA family lipoprotein |
| BCAL3205 |  | hypothetical protein |
| BCAL3255 |  | putative DedA family protein |
| BCAL3257 | *miaA* | tRNA delta(2)-isopentenylpyrophosphate transf |
| BCAL3262 | *hda* | DnaA regulatory inactivator Hda |
| BCAL3263 |  | hypothetical protein |
| BCAL3264 |  | putative polynucleotide adenylyltr |
| BCAL3269 | *dnaJ* | putative DnaJ chaperone protein |
| BCAL3270 | *dnaK* | molecular chaperone DnaK |
| BCAL3272 |  | putative heat shock protein |
| BCAL3276 | *ppnK* | NAD(+)/NADH kinase family protein |
| BCAL3277 |  | putative RecN DNA repair protein |
| BCAL3278 | *glnE* | putative glutamate-ammonia-ligase adenylyltr |
| BCAL3281 | *tldD* | putative DNA gyrase control protein |
| BCAL3290 | *glcF* | glycolate oxidase iron-sulfur subunit |
| BCAL3292 |  | pyrroline-5-carboxylate reductase |
| BCAL3296 | *ubiA* | 4-hydroxybenzoate octaprenyltransferase |
| BCAL3302 | *recG* | ATP-dependent DNA helicase RecG |
| BCAL3304 | *tgt* | putative queuine tRNA-ribosyltransferase |
| BCAL3305 | *yajC* | preprotein translocase subunit YajC |
| BCAL3306 | *secD* | preprotein translocase subunit SecD |
| BCAL3307 | *secF* | preprotein translocase subunit SecF |
| BCAL3329 | *mnmA* | tRNA-specific 2-thiouridylase MnmA |
| BCAL3331 |  | putative glutathione S-transferase |
| BCAL3334 |  | putative nitrogen regulation-related protein |
| BCAL3336 | *purH* | bif phosphoribosylaminoimidazolecarboxam formyltr |
| BCAL3337 | *ruvC* | Holliday junction resolvase |
| BCAL3338 | *ruvA* | Holliday junction DNA helicase RuvA |
| BCAL3339 | *ruvB* | Holliday junction DNA helicase RuvB |
| BCAL3344 | *tyrZ* | tyrosyl-tRNA synthetase |
| BCAL3345 | *anmK* | anhydro-N-acetylmuramic acid kinase |
| BCAL3346 |  | iron-sulfur cluster insertion protein ErpA |
| BCAL3347 | *rpsI* | 30S ribosomal protein S9 |
| BCAL3348 | *rplM* | 50S ribosomal protein L13 |
| BCAL3349 |  | putative OsmC-like protein |
| BCAL3351 | *pyrC* | dihydroorotase |
| BCAL3361 | *purB* | adenylosuccinate lyase |
| BCAL3369 |  | hypothetical protein |
| BCAL3370 | *proA* | gamma-glutamyl phosphate reductase |
| BCAL3371 | *holA* | DNA polymerase III subunit delta |
| BCAL3372 |  | putative lipoprotein |
| BCAL3373 | *leuS* | leucyl-tRNA synthetase |
| BCAL3376 | *dapB* | dihydrodipicolinate reductase |
| BCAL3377 | *omlA* | putative outer membrane protein |
| BCAL3378 | *fur* | ferric uptake regulator |
| BCAL3388 | *gapA* | glyceraldehyde 3-phosphate dehydrogenase 1 |
| BCAL3389 | *tatA* | transketolase |
| BCAL3392 |  | 16S ribosomal RNA methyltransferase RsmE |
| BCAL3397 |  | putative phosphatidylglycerophosphatase |
| BCAL3398 |  | putative competence-damaged related protein |
| BCAL3400 | *pyrF* | orotidine 5'-phosphate decarboxylase |
| BCAL3412 | *mtgA* | monofunct biosynth peptidoglycan transglyc |
| BCAL3413 | *aroE* | shikimate 5-dehydrogenase |
| BCAL3414 |  | putative exoribonuclease II |
| BCAL3416 | *mpl* | UDP-N-acetylm:L-ala-gamma-D-g-meso-iaminop l |
| BCAL3418 |  | putative thioredoxin protein |
| BCAL3420 | *accB* | acetyl-CoA carbox biotin carbox carrier prot sub |
| BCAL3421 | *accC* | acetyl-CoA carbox biotin carboxylase sub |
| BCAL3425 |  | putative sugar kinase |
| BCAL3428 | *nrdB* | ribonucleotide-diphosphate reductase sub beta |
| BCAL3429 |  | ribonucleotide-diphosphate reductase sub alpha |
| BCAL3430 | *ampD* | N-acetyl-anhydromuranmyl-L-alanine amidase |
| BCAL3432 |  | cytochrome c assembly protein |
| BCAL3433 | *ffh* | signal recognition particle protein |
| BCAL3436 | *proS* | prolyl-tRNA synthetase |
| BCAL3437 |  | dinucleoside polyphosphate hydrolase |
| BCAL3439 | *proB* | gamma-glutamyl kinase |
| BCAL3440 | *obgE* | GTPase ObgE |
| BCAL3441 | *rpmA* | 50S ribosomal protein L27 |
| BCAL3443 | *ispB* | octaprenyl-diphosphate synthase |
| BCAL3447 | *gspO* | type IV prepilin leader peptide type M1 |
| BCAL3448 | *coaE* | dephospho-CoA kinase |
| BCAL3450 |  | NUDIX hydrolase |
| BCAL3452 | *argJ* | bif ornithine acetyltr/N-acetylglutamate synt prot |
| BCAL3453 | *secA* | preprotein translocase subunit SecA |
| BCAL3455 | *lpxC* | UDP-3-O-[3-hydroxymyristoyl] N-acetylgluc deacet |
| BCAL3456 |  | putative thioredoxin reductase |
| BCAL3457 | *ftsZ* | cell division protein FtsZ |
| BCAL3458 | *ftsA* | cell division protein FtsA |
| BCAL3460 | *ddl* | D-alanine--D-alanine ligase |
| BCAL3461 | *murC* | UDP-N-acetylmuramate--L-alanine ligase |
| BCAL3462 | *murG* | undecaprenyldiph-muramoylpentap beta-N- acetylgl |
| BCAL3463 | *ftsW* | cell division protein FtsW |
| BCAL3464 | *murD* | UDP-N-acetylmur-L-alanyl-D-glutamate synth |
| BCAL3467 | *murE* | UDP-N-acetylmuramoyla-D-glut--2, 6-diaminop lig |
| BCAL3468 | *ftsI* | peptidoglycan synthetase FtsI |
| BCAL3470 | *mraW* | S-adenosyl-methyltransferase MraW |
| BCAL3472 |  | hypothetical protein |
| BCAL3474 | *fadD* | acyl-CoA synthetase |
| BCAM0478 | *glmS2* | glucosa--fructe-6-phosph aminotr [isomerizing] 2 |
| BCAM0548 | *groEL* | chaperonin GroEL |
| BCAM0732 |  | cysteine desulfurase |
| BCAM0746 | *argG* | argininosuccinate synthase |
| BCAM0829 |  | riboflavin synthase subunit alpha |
| BCAM0915 | *rpsU* | 30S ribosomal protein S21 |
| BCAM1224 | *glmS4* | glucosa--fruct-6-phosph aminotr [isomerizing] 4 |
| BCAM1944 |  | 2-oxoacid dehydrogenase subunit E1 |
| BCAM2076 | *lysA* | diaminopimelate decarboxylase |
| BCAM2216 |  | hypothetical protein |
| BCAS0609 |  | putative electron transport protein |
| BCAS0637 | *groEL* | chaperonin GroEL |
| BCAS0711 |  | 2-oxoacid dehydrogenase subunit E1 |
