# Supplementary material for: High Confidence Prediction of Essential Genes in Burkholderia Cenocepacia
Source: PLoS One. 2012 Jun 29;7(6):e40064. doi: 10.1371/journal.pone.0040064 (PMC3386938; doi:10.1371/journal.pone.0040064)
Supplement: Table S3 — Burkholderiales core genome DEG homologs. (DOC) [file pone.0040064.s006.doc]

**Table S3. *Burkholderiales* core genome DEG homologs.**

| **Locus tag** | **Best DEG hit** | **Organism of best DEG hit** |
| --- | --- | --- |
| BCAL0227 | DEG10030052 | Vibrio cholerae N16961 |
| BCAL1262 | DEG10130344 | Acinetobacter baylyi ADP1 |
| BCAL3429 | DEG10150228 | Pseudomonas aeruginosa UCBPP-PA14 |
| BCAL1515 | DEG10150205 | Pseudomonas aeruginosa UCBPP-PA14 |
| BCAL1919 | DEG10110149 | Salmonella typhimurium LT2 |
| BCAL3453 | DEG10190021 | Escherichia coli MG1655 II |
| BCAL0825 | DEG10020067 | Staphylococcus aureus N315 |
| BCAL2017 | DEG10050452 | Haemophilus influenzae Rd KW20 |
| BCAL0231 | DEG10190231 | Escherichia coli MG1655 II |
| BCAL2957 | DEG10150117 | Pseudomonas aeruginosa UCBPP-PA14 |
| BCAL2739 | DEG10190231 | Escherichia coli MG1655 II |
| BCAL1416 | DEG10180418 | Escherichia coli MG1655 I |
| BCAL2724 | DEG10150271 | Pseudomonas aeruginosa UCBPP-PA14 |
| BCAL1994 | DEG10160164 | Salmonella enterica serovar Typhi |
| BCAL1448 | DEG10150075 | Pseudomonas aeruginosa UCBPP-PA14 |
| BCAM1944 | DEG10160025 | Salmonella enterica serovar Typhi |
| BCAL2409 | DEG10190041 | Escherichia coli MG1655 II |
| BCAL3373 | DEG10130395 | Acinetobacter baylyi ADP1 |
| BCAL0421 | DEG10160277 | Salmonella enterica serovar Typhi |
| BCAL2348 | DEG10180469 | Escherichia coli MG1655 I |
| BCAL3270 | DEG10160001 | Salmonella enterica serovar Typhi |
| BCAL3389 | DEG10050368 | Haemophilus influenzae Rd KW20 |
| BCAL1481 | DEG10130387 | Acinetobacter baylyi ADP1 |
| BCAL0034 | DEG10130026 | Acinetobacter baylyi ADP1 |
| BCAL2096 | DEG10190139 | Escherichia coli MG1655 II |
| BCAL1507 | DEG10150286 | Pseudomonas aeruginosa UCBPP-PA14 |
| BCAL1003 | DEG10130313 | Acinetobacter baylyi ADP1 |
| BCAL2867 | DEG10130313 | Acinetobacter baylyi ADP1 |
| BCAL2646 | DEG10050455 | Haemophilus influenzae Rd KW20 |
| BCAL0880 | DEG10130100 | Acinetobacter baylyi ADP1 |
| BCAL0024 | DEG10120264 | Francisella novicida U112 |
| BCAL0036 | DEG10130028 | Acinetobacter baylyi ADP1 |
| BCAL1267 | DEG10130342 | Acinetobacter baylyi ADP1 |
| BCAL2993 | DEG10050582 | Haemophilus influenzae Rd KW20 |
| BCAL2224 | DEG10130294 | Acinetobacter baylyi ADP1 |
| BCAM0746 | DEG10130167 | Acinetobacter baylyi ADP1 |
| BCAL2061 | DEG10050104 | Haemophilus influenzae Rd KW20 |
| BCAL2181 | DEG10150107 | Pseudomonas aeruginosa UCBPP-PA14 |
| BCAL3436 | DEG10150236 | Pseudomonas aeruginosa UCBPP-PA14 |
| BCAL3146 | DEG10160337 | Salmonella enterica serovar Typhi |
| BCAL2950 | DEG10030375 | Vibrio cholerae N16961 |
| BCAL0611 | DEG10150333 | Pseudomonas aeruginosa UCBPP-PA14 |
| BCAL3010 | DEG10190252 | Escherichia coli MG1655 II |
| BCAL0982 | DEG10160120 | Salmonella enterica serovar Typhi |
| BCAL2888 | DEG10160120 | Salmonella enterica serovar Typhi |
| BCAL2063 | DEG10130475 | Acinetobacter baylyi ADP1 |
| BCAL3336 | DEG10130291 | Acinetobacter baylyi ADP1 |
| BCAL2194 | DEG10050129 | Haemophilus influenzae Rd KW20 |
| BCAL2341 | DEG10120378 | Francisella novicida U112 |
| BCAL1468 | DEG10110052 | Salmonella typhimurium LT2 |
| BCAL3421 | DEG10150294 | Pseudomonas aeruginosa UCBPP-PA14 |
| BCAL2455 | DEG10030490 | Vibrio cholerae N16961 |
| BCAL1413 | DEG10030209 | Vibrio cholerae N16961 |
| BCAL3361 | DEG10150144 | Pseudomonas aeruginosa UCBPP-PA14 |
| BCAL1995 | DEG10110034 | Salmonella typhimurium LT2 |
| BCAL1901 | DEG10030039 | Vibrio cholerae N16961 |
| BCAL2198 | DEG10180388 | Escherichia coli MG1655 I |
| BCAL2454 | DEG10180449 | Escherichia coli MG1655 I |
| BCAL2207 | DEG10110014 | Salmonella typhimurium LT2 |
| BCAL0219 | DEG10180508 | Escherichia coli MG1655 I |
| BCAL0232 | DEG10180508 | Escherichia coli MG1655 I |
| BCAL2417 | DEG10190073 | Escherichia coli MG1655 II |
| BCAL0485 | DEG10150264 | Pseudomonas aeruginosa UCBPP-PA14 |
| BCAL1449 | DEG10050427 | Haemophilus influenzae Rd KW20 |
| BCAL1486 | DEG10190117 | Escherichia coli MG1655 II |
| BCAL2190 | DEG10110164 | Salmonella typhimurium LT2 |
| BCAL2179 | DEG10010237 | Bacillus subtilis 168 |
| BCAL2638 | DEG10130043 | Acinetobacter baylyi ADP1 |
| BCAL0145 | DEG10100518 | Mycobacterium tuberculosis H37Rv |
| BCAL3433 | DEG10160183 | Salmonella enterica serovar Typhi |
| BCAL3197 | DEG10150275 | Pseudomonas aeruginosa UCBPP-PA14 |
| BCAL3461 | DEG10150250 | Pseudomonas aeruginosa UCBPP-PA14 |
| BCAL3030 | DEG10190075 | Escherichia coli MG1655 II |
| BCAL2083 | DEG10150100 | Pseudomonas aeruginosa UCBPP-PA14 |
| BCAL2456 | DEG10050464 | Haemophilus influenzae Rd KW20 |
| BCAL1941 | DEG10030066 | Vibrio cholerae N16961 |
| BCAL0509 | DEG10190171 | Escherichia coli MG1655 II |
| BCAL1884 | DEG10120242 | Francisella novicida U112 |
| BCAL2339 | DEG10120376 | Francisella novicida U112 |
| BCAL3428 | DEG10150229 | Pseudomonas aeruginosa UCBPP-PA14 |
| BCAL2359 | DEG10100485 | Mycobacterium tuberculosis H37Rv |
| BCAL3370 | DEG10130093 | Acinetobacter baylyi ADP1 |
| BCAL3049 | DEG10130169 | Acinetobacter baylyi ADP1 |
| BCAL1873 | DEG10030539 | Vibrio cholerae N16961 |
| BCAL2146 | DEG10130174 | Acinetobacter baylyi ADP1 |
| BCAL3281 | DEG10050603 | Haemophilus influenzae Rd KW20 |
| BCAL2994 | DEG10150317 | Pseudomonas aeruginosa UCBPP-PA14 |
| BCAL2839 | DEG10150018 | Pseudomonas aeruginosa UCBPP-PA14 |
| BCAL0310 | DEG10130110 | Acinetobacter baylyi ADP1 |
| BCAL2389 | DEG10130292 | Acinetobacter baylyi ADP1 |
| BCAL0387 | DEG10180201 | Escherichia coli MG1655 I |
| BCAL0484 | DEG10130126 | Acinetobacter baylyi ADP1 |
| BCAL2333 | DEG10120370 | Francisella novicida U112 |
| BCAL3344 | DEG10120232 | Francisella novicida U112 |
| BCAL0876 | DEG10150313 | Pseudomonas aeruginosa UCBPP-PA14 |
| BCAL2338 | DEG10120375 | Francisella novicida U112 |
| BCAL3304 | DEG10050107 | Haemophilus influenzae Rd KW20 |
| BCAL0612 | DEG10190261 | Escherichia coli MG1655 II |
| BCAL0254 | DEG10030517 | Vibrio cholerae N16961 |
| BCAL0399 | DEG10130045 | Acinetobacter baylyi ADP1 |
| BCAL2357 | DEG10130393 | Acinetobacter baylyi ADP1 |
| BCAL0312 | DEG10130112 | Acinetobacter baylyi ADP1 |
| BCAL2841 | DEG10190170 | Escherichia coli MG1655 II |
| BCAL3416 | DEG10050055 | Haemophilus influenzae Rd KW20 |
| BCAL0482 | DEG10030073 | Vibrio cholerae N16961 |
| BCAL0903 | DEG10160288 | Salmonella enterica serovar Typhi |
| BCAL2150 | DEG10130212 | Acinetobacter baylyi ADP1 |
| BCAL3458 | DEG10150249 | Pseudomonas aeruginosa UCBPP-PA14 |
| BCAL3339 | DEG10160083 | Salmonella enterica serovar Typhi |
| BCAL3452 | DEG10130108 | Acinetobacter baylyi ADP1 |
| BCAL0869 | DEG10130192 | Acinetobacter baylyi ADP1 |
| BCAL0374 | DEG10120325 | Francisella novicida U112 |
| BCAL2126 | DEG10030428 | Vibrio cholerae N16961 |
| BCAL2109 | DEG10050571 | Haemophilus influenzae Rd KW20 |
| BCAL2676 | DEG10050625 | Haemophilus influenzae Rd KW20 |
| BCAL0904 | DEG10150003 | Pseudomonas aeruginosa UCBPP-PA14 |
| BCAL1269 | DEG10160225 | Salmonella enterica serovar Typhi |
| BCAL1883 | DEG10190144 | Escherichia coli MG1655 II |
| BCAL0479 | DEG10030196 | Vibrio cholerae N16961 |
| BCAL0041 | DEG10160267 | Salmonella enterica serovar Typhi |
| BCAL0800 | DEG10130356 | Acinetobacter baylyi ADP1 |
| BCAL2332 | DEG10120369 | Francisella novicida U112 |
| BCAL0493 | DEG10130091 | Acinetobacter baylyi ADP1 |
| BCAL1506 | DEG10190183 | Escherichia coli MG1655 II |
| BCAL2408 | DEG10190077 | Escherichia coli MG1655 II |
| BCAL0428 | DEG10180543 | Escherichia coli MG1655 I |
| BCAL1467 | DEG10050092 | Haemophilus influenzae Rd KW20 |
| BCAL1926 | DEG10130039 | Acinetobacter baylyi ADP1 |
| BCAL2148 | DEG10150105 | Pseudomonas aeruginosa UCBPP-PA14 |
| BCAL3306 | DEG10190047 | Escherichia coli MG1655 II |
| BCAL0956 | DEG10170153 | Staphylococcus aureus NCTC 8325 |
| BCAL0996 | DEG10120300 | Francisella novicida U112 |
| BCAL2874 | DEG10120300 | Francisella novicida U112 |
| BCAL0953 | DEG10160189 | Salmonella enterica serovar Typhi |
| BCAL0679 | DEG10170056 | Staphylococcus aureus NCTC 8325 |
| BCAL2101 | DEG10130316 | Acinetobacter baylyi ADP1 |
| BCAL0710 | DEG10150066 | Pseudomonas aeruginosa UCBPP-PA14 |
| BCAL2764 | DEG10110070 | Salmonella typhimurium LT2 |
| BCAL3035 | DEG10130147 | Acinetobacter baylyi ADP1 |
| BCAL0423 | DEG10190255 | Escherichia coli MG1655 II |
| BCAL1270 | DEG10110203 | Salmonella typhimurium LT2 |
| BCAL3351 | DEG10130166 | Acinetobacter baylyi ADP1 |
| BCAL1899 | DEG10190054 | Escherichia coli MG1655 II |
| BCAL3135 | DEG10050317 | Haemophilus influenzae Rd KW20 |
| BCAL0237 | DEG10030534 | Vibrio cholerae N16961 |
| BCAL2103 | DEG10130124 | Acinetobacter baylyi ADP1 |
| BCAL0558 | DEG10130266 | Acinetobacter baylyi ADP1 |
| BCAL2710 | DEG10130450 | Acinetobacter baylyi ADP1 |
| BCAL0613 | DEG10030284 | Vibrio cholerae N16961 |
| BCAL3468 | DEG10160011 | Salmonella enterica serovar Typhi |
| BCAL2955 | DEG10130326 | Acinetobacter baylyi ADP1 |
| BCAL0264 | DEG10130153 | Acinetobacter baylyi ADP1 |
| BCAL1485 | DEG10190118 | Escherichia coli MG1655 II |
| BCAL2390 | DEG10150007 | Pseudomonas aeruginosa UCBPP-PA14 |
| BCAL3329 | DEG10150145 | Pseudomonas aeruginosa UCBPP-PA14 |
| BCAL1517 | DEG20060091 | Homo sapiens |
| BCAL0375 | DEG10150280 | Pseudomonas aeruginosa UCBPP-PA14 |
| BCAL0260 | DEG10190205 | Escherichia coli MG1655 II |
| BCAL2208 | DEG10050441 | Haemophilus influenzae Rd KW20 |
| BCAL2935 | DEG10150133 | Pseudomonas aeruginosa UCBPP-PA14 |
| BCAL3455 | DEG10030474 | Vibrio cholerae N16961 |
| BCAL2085 | DEG10150099 | Pseudomonas aeruginosa UCBPP-PA14 |
| BCAL2767 | DEG10130195 | Acinetobacter baylyi ADP1 |
| BCAL2186 | DEG10150122 | Pseudomonas aeruginosa UCBPP-PA14 |
| BCAL3388 | DEG10100232 | Mycobacterium tuberculosis H37Rv |
| BCAL2838 | DEG10050633 | Haemophilus influenzae Rd KW20 |
| BCAL0318 | DEG10130468 | Acinetobacter baylyi ADP1 |
| BCAL0280 | DEG10130454 | Acinetobacter baylyi ADP1 |
| BCAL2167 | DEG10150232 | Pseudomonas aeruginosa UCBPP-PA14 |
| BCAL0460 | DEG10030715 | Vibrio cholerae N16961 |
| BCAL0992 | DEG10180178 | Escherichia coli MG1655 I |
| BCAL2878 | DEG10180178 | Escherichia coli MG1655 I |
| BCAL1516 | DEG10150204 | Pseudomonas aeruginosa UCBPP-PA14 |
| BCAL2934 | DEG10130329 | Acinetobacter baylyi ADP1 |
| BCAL0426 | DEG10120008 | Francisella novicida U112 |
| BCAL3467 | DEG10150255 | Pseudomonas aeruginosa UCBPP-PA14 |
| BCAL1625 | DEG10050204 | Haemophilus influenzae Rd KW20 |
| BCAL2726 | DEG10030025 | Vibrio cholerae N16961 |
| BCAL3440 | DEG10130308 | Acinetobacter baylyi ADP1 |
| BCAL0496 | DEG10130151 | Acinetobacter baylyi ADP1 |
| BCAL0462 | DEG10110077 | Salmonella typhimurium LT2 |
| BCAL2092 | DEG10120124 | Francisella novicida U112 |
| BCAL2337 | DEG10120374 | Francisella novicida U112 |
| BCAL0736 | DEG10020092 | Staphylococcus aureus N315 |
| BCAL0993 | DEG10030392 | Vibrio cholerae N16961 |
| BCAL2877 | DEG10030392 | Vibrio cholerae N16961 |
| BCAL0873 | DEG10150312 | Pseudomonas aeruginosa UCBPP-PA14 |
| BCAL2091 | DEG10150094 | Pseudomonas aeruginosa UCBPP-PA14 |
| BCAL2916 | DEG10050418 | Haemophilus influenzae Rd KW20 |
| BCAL3439 | DEG10130307 | Acinetobacter baylyi ADP1 |
| BCAL0401 | DEG10180517 | Escherichia coli MG1655 I |
| BCAL2768 | DEG10190272 | Escherichia coli MG1655 II |
| BCAL3050 | DEG10150057 | Pseudomonas aeruginosa UCBPP-PA14 |
| BCAL0240 | DEG10150038 | Pseudomonas aeruginosa UCBPP-PA14 |
| BCAL2078 | DEG10150103 | Pseudomonas aeruginosa UCBPP-PA14 |
| BCAL2644 | DEG10110125 | Salmonella typhimurium LT2 |
| BCAL1996 | DEG10050238 | Haemophilus influenzae Rd KW20 |
| BCAL2212 | DEG10010172 | Bacillus subtilis 168 |
| BCAL3257 | DEG10050031 | Haemophilus influenzae Rd KW20 |
| BCAL0035 | DEG10030560 | Vibrio cholerae N16961 |
| BCAL0397 | DEG10130296 | Acinetobacter baylyi ADP1 |
| BCAL0422 | DEG10160276 | Salmonella enterica serovar Typhi |
| BCAL0478 | DEG10030195 | Vibrio cholerae N16961 |
| BCAL0814 | DEG10120209 | Francisella novicida U112 |
| BCAL0911 | DEG10080042 | Helicobacter pylori 26695 |
| BCAL1611 | DEG10130186 | Acinetobacter baylyi ADP1 |
| BCAL2110 | DEG10030067 | Vibrio cholerae N16961 |
| BCAL0259 | DEG10050287 | Haemophilus influenzae Rd KW20 |
| BCAL1006 | DEG10130311 | Acinetobacter baylyi ADP1 |
| BCAL2864 | DEG10130311 | Acinetobacter baylyi ADP1 |
| BCAL3269 | DEG10170213 | Staphylococcus aureus NCTC 8325 |
| BCAL0223 | DEG10030048 | Vibrio cholerae N16961 |
| BCAL2959 | DEG10150116 | Pseudomonas aeruginosa UCBPP-PA14 |
| BCAL3460 | DEG10110011 | Salmonella typhimurium LT2 |
| BCAL0314 | DEG10130465 | Acinetobacter baylyi ADP1 |
| BCAL0317 | DEG10130467 | Acinetobacter baylyi ADP1 |
| BCAL3376 | DEG10160006 | Salmonella enterica serovar Typhi |
| BCAL3462 | DEG10190014 | Escherichia coli MG1655 II |
| BCAL3470 | DEG10160009 | Salmonella enterica serovar Typhi |
| BCAL2079 | DEG10190039 | Escherichia coli MG1655 II |
| BCAL0793 | DEG10160290 | Salmonella enterica serovar Typhi |
| BCAL2087 | DEG10150097 | Pseudomonas aeruginosa UCBPP-PA14 |
| BCAL3203 | DEG10030352 | Vibrio cholerae N16961 |
| BCAL3443 | DEG10160231 | Salmonella enterica serovar Typhi |
| BCAL2084 | DEG10160040 | Salmonella enterica serovar Typhi |
| BCAL2425 | DEG10050614 | Haemophilus influenzae Rd KW20 |
| BCAL0787 | DEG10130183 | Acinetobacter baylyi ADP1 |
| BCAL0994 | DEG10030391 | Vibrio cholerae N16961 |
| BCAL2876 | DEG10030391 | Vibrio cholerae N16961 |
| BCAL3457 | DEG10110013 | Salmonella typhimurium LT2 |
| BCAL2089 | DEG10160035 | Salmonella enterica serovar Typhi |
| BCAL0301 | DEG10050394 | Haemophilus influenzae Rd KW20 |
| BCAL0409 | DEG20020162 | Caenorhabditis elegans |
| BCAL0466 | DEG10150004 | Pseudomonas aeruginosa UCBPP-PA14 |
| BCAL1275 | DEG10030155 | Vibrio cholerae N16961 |
| BCAL1865 | DEG10050080 | Haemophilus influenzae Rd KW20 |
| BCAL2762 | DEG10160162 | Salmonella enterica serovar Typhi |
| BCAL1504 | DEG10180218 | Escherichia coli MG1655 I |
| BCAL2081 | DEG10050331 | Haemophilus influenzae Rd KW20 |
| BCAL1276 | DEG10110029 | Salmonella typhimurium LT2 |
| BCAL2117 | DEG10030139 | Vibrio cholerae N16961 |
| BCAL0991 | DEG10120295 | Francisella novicida U112 |
| BCAL2879 | DEG10120295 | Francisella novicida U112 |
| BCAL0398 | DEG10130295 | Acinetobacter baylyi ADP1 |
| BCAL3110 | DEG10160283 | Salmonella enterica serovar Typhi |
| BCAL2180 | DEG10130246 | Acinetobacter baylyi ADP1 |
| BCAL2412 | DEG20050462 | Mus musculus |
| BCAL0234 | DEG10030537 | Vibrio cholerae N16961 |
| BCAL0894 | DEG10190006 | Escherichia coli MG1655 II |
| BCAL0739 | DEG10050256 | Haemophilus influenzae Rd KW20 |
| BCAL3463 | DEG10030480 | Vibrio cholerae N16961 |
| BCAL0329 | DEG10080311 | Helicobacter pylori 26695 |
| BCAL3296 | DEG10190280 | Escherichia coli MG1655 II |
| BCAL0246 | DEG10150044 | Pseudomonas aeruginosa UCBPP-PA14 |
| BCAL1254 | DEG10180057 | Escherichia coli MG1655 I |
| BCAL2926 | DEG10110150 | Salmonella typhimurium LT2 |
| BCAL0505 | DEG10180554 | Escherichia coli MG1655 I |
| BCAL0897 | DEG10030079 | Vibrio cholerae N16961 |
| BCAL1509 | DEG10050460 | Haemophilus influenzae Rd KW20 |
| BCAL3053 | DEG10100229 | Mycobacterium tuberculosis H37Rv |
| BCAL1556 | DEG10160201 | Salmonella enterica serovar Typhi |
| BCAL2090 | DEG10130264 | Acinetobacter baylyi ADP1 |
| BCAL2343 | DEG10120380 | Francisella novicida U112 |
| BCAL3090 | DEG10030084 | Vibrio cholerae N16961 |
| BCAL0957 | DEG10100143 | Mycobacterium tuberculosis H37Rv |
| BCAL2104 | DEG10130249 | Acinetobacter baylyi ADP1 |
| BCAL1651 | DEG10190282 | Escherichia coli MG1655 II |
| BCAL0221 | DEG10030046 | Vibrio cholerae N16961 |
| BCAL2157 | DEG10190146 | Escherichia coli MG1655 II |
| BCAL2954 | DEG10130259 | Acinetobacter baylyi ADP1 |
| BCAL0026 | DEG10100612 | Mycobacterium tuberculosis H37Rv |
| BCAL3371 | DEG10150067 | Pseudomonas aeruginosa UCBPP-PA14 |
| BCAL3276 | DEG10130261 | Acinetobacter baylyi ADP1 |
| BCAL3345 | DEG10050254 | Haemophilus influenzae Rd KW20 |
| BCAL0325 | DEG10050087 | Haemophilus influenzae Rd KW20 |
| BCAL2088 | DEG10130197 | Acinetobacter baylyi ADP1 |
| BCAL2651 | DEG10030121 | Vibrio cholerae N16961 |
| BCAL0030 | DEG10030565 | Vibrio cholerae N16961 |
| BCAL2153 | DEG10180098 | Escherichia coli MG1655 I |
| BCAL2782 | DEG10150231 | Pseudomonas aeruginosa UCBPP-PA14 |
| BCAL2358 | DEG10130394 | Acinetobacter baylyi ADP1 |
| BCAL3464 | DEG10130040 | Acinetobacter baylyi ADP1 |
| BCAL0160 | DEG10050604 | Haemophilus influenzae Rd KW20 |
| BCAL0230 | DEG10130145 | Acinetobacter baylyi ADP1 |
| BCAL0808 | DEG10050415 | Haemophilus influenzae Rd KW20 |
| BCAL0235a | DEG10190228 | Escherichia coli MG1655 II |
| BCAL2197 | DEG10030159 | Vibrio cholerae N16961 |
| BCAL0222 | DEG10150030 | Pseudomonas aeruginosa UCBPP-PA14 |
| BCAL2622 | DEG10190292 | Escherichia coli MG1655 II |
| BCAL0302 | DEG10050393 | Haemophilus influenzae Rd KW20 |
| BCAL2761 | DEG10110058 | Salmonella typhimurium LT2 |
| BCAL2947 | DEG10160105 | Salmonella enterica serovar Typhi |
| BCAL0373 | DEG10110104 | Salmonella typhimurium LT2 |
| BCAL3200 | DEG10030354 | Vibrio cholerae N16961 |
| BCAL0258 | DEG10130410 | Acinetobacter baylyi ADP1 |
| BCAL1874 | DEG10130177 | Acinetobacter baylyi ADP1 |
| BCAL2346 | DEG10050225 | Haemophilus influenzae Rd KW20 |
| BCAL0388 | DEG10030498 | Vibrio cholerae N16961 |
| BCAL0709 | DEG10050012 | Haemophilus influenzae Rd KW20 |
| BCAL0229 | DEG10190233 | Escherichia coli MG1655 II |
| BCAL1004 | DEG10030495 | Vibrio cholerae N16961 |
| BCAL2866 | DEG10030495 | Vibrio cholerae N16961 |
| BCAL3307 | DEG10120245 | Francisella novicida U112 |
| BCAL0465 | DEG10130031 | Acinetobacter baylyi ADP1 |
| BCAL0907 | DEG10110045 | Salmonella typhimurium LT2 |
| BCAL0244 | DEG10150042 | Pseudomonas aeruginosa UCBPP-PA14 |
| BCAL3348 | DEG10130371 | Acinetobacter baylyi ADP1 |
| BCAL0249 | DEG10150046 | Pseudomonas aeruginosa UCBPP-PA14 |
| BCAL0895 | DEG10110003 | Salmonella typhimurium LT2 |
| BCAL2336 | DEG10120373 | Francisella novicida U112 |
| BCAL0795 | DEG10150013 | Pseudomonas aeruginosa UCBPP-PA14 |
| BCAL1255 | DEG10150188 | Pseudomonas aeruginosa UCBPP-PA14 |
| BCAL2912 | DEG10150011 | Pseudomonas aeruginosa UCBPP-PA14 |
| BCAL1274 | DEG10160271 | Salmonella enterica serovar Typhi |
| BCAL1887 | DEG10150082 | Pseudomonas aeruginosa UCBPP-PA14 |
| BCAL3472 | DEG10130283 | Acinetobacter baylyi ADP1 |
| BCAL3012 | DEG10190251 | Escherichia coli MG1655 II |
| BCAL0299 | DEG10100053 | Mycobacterium tuberculosis H37Rv |
| BCAL0316 | DEG10130466 | Acinetobacter baylyi ADP1 |
| BCAL1964 | DEG10150132 | Pseudomonas aeruginosa UCBPP-PA14 |
| BCAL2172 | DEG10050507 | Haemophilus influenzae Rd KW20 |
| BCAL0147 | DEG20060002 | Homo sapiens |
| BCAL0251 | DEG10190212 | Escherichia coli MG1655 II |
| BCAL1852 | DEG10130489 | Acinetobacter baylyi ADP1 |
| BCAL2077 | DEG10180050 | Escherichia coli MG1655 I |
| BCAL1962 | DEG10020032 | Staphylococcus aureus N315 |
| BCAL3051 | DEG10150058 | Pseudomonas aeruginosa UCBPP-PA14 |
| BCAL3292 | DEG10130377 | Acinetobacter baylyi ADP1 |
| BCAL2355 | DEG10130403 | Acinetobacter baylyi ADP1 |
| BCAL0241 | DEG10030530 | Vibrio cholerae N16961 |
| BCAL2196 | DEG10150079 | Pseudomonas aeruginosa UCBPP-PA14 |
| BCAL1266 | DEG10160227 | Salmonella enterica serovar Typhi |
| BCAL1925 | DEG10050039 | Haemophilus influenzae Rd KW20 |
| BCAL3263 | DEG10130076 | Acinetobacter baylyi ADP1 |
| BCAL0248 | DEG10030523 | Vibrio cholerae N16961 |
| BCAL2154 | DEG10190057 | Escherichia coli MG1655 II |
| BCAL2921 | DEG10160339 | Salmonella enterica serovar Typhi |
| BCAL0806 | DEG20051918 | Mus musculus |
| BCAL0253 | DEG10120072 | Francisella novicida U112 |
| BCAL0027 | DEG10100611 | Mycobacterium tuberculosis H37Rv |
| BCAM2076 | DEG10080050 | Helicobacter pylori 26695 |
| BCAL0257 | DEG10150051 | Pseudomonas aeruginosa UCBPP-PA14 |
| BCAL3347 | DEG10030118 | Vibrio cholerae N16961 |
| BCAL1005 | DEG10190150 | Escherichia coli MG1655 II |
| BCAL1900 | DEG10150321 | Pseudomonas aeruginosa UCBPP-PA14 |
| BCAL2865 | DEG10190150 | Escherichia coli MG1655 II |
| BCAL3278 | DEG10100353 | Mycobacterium tuberculosis H37Rv |
| BCAL2759 | DEG10050027 | Haemophilus influenzae Rd KW20 |
| BCAL3346 | DEG10150028 | Pseudomonas aeruginosa UCBPP-PA14 |
| BCAL3392 | DEG10050117 | Haemophilus influenzae Rd KW20 |
| BCAL3437 | DEG10050324 | Haemophilus influenzae Rd KW20 |
| BCAL0743 | DEG10030545 | Vibrio cholerae N16961 |
| BCAL0798 | DEG10190100 | Escherichia coli MG1655 II |
| BCAL2678 | DEG10030501 | Vibrio cholerae N16961 |
| BCAL1922 | DEG10030218 | Vibrio cholerae N16961 |
| BCAL1463 | DEG10050112 | Haemophilus influenzae Rd KW20 |
| BCAL2925 | DEG10030113 | Vibrio cholerae N16961 |
| BCAL3447 | DEG10030487 | Vibrio cholerae N16961 |
| BCAL0233 | DEG10030538 | Vibrio cholerae N16961 |
| BCAL0809 | DEG10170072 | Staphylococcus aureus NCTC 8325 |
| BCAL0988 | DEG10110071 | Salmonella typhimurium LT2 |
| BCAL2016 | DEG10050221 | Haemophilus influenzae Rd KW20 |
| BCAL2184 | DEG10180608 | Escherichia coli MG1655 I |
| BCAL2882 | DEG10110071 | Salmonella typhimurium LT2 |
| BCAL3448 | DEG10180027 | Escherichia coli MG1655 I |
| BCAM0829 | DEG10050581 | Haemophilus influenzae Rd KW20 |
| BCAL0261 | DEG10050289 | Haemophilus influenzae Rd KW20 |
| BCAL0827 | DEG10190284 | Escherichia coli MG1655 II |
| BCAL0319 | DEG10130067 | Acinetobacter baylyi ADP1 |
| BCAL2342 | DEG10120379 | Francisella novicida U112 |
| BCAL0487 | DEG20051212 | Mus musculus |
| BCAL2915 | DEG10030078 | Vibrio cholerae N16961 |
| BCAL1945 | DEG10030062 | Vibrio cholerae N16961 |
| BCAL2920 | DEG20052048 | Mus musculus |
| BCAL1484 | DEG10030597 | Vibrio cholerae N16961 |
| BCAL1473 | DEG10050265 | Haemophilus influenzae Rd KW20 |
| BCAL0238 | DEG10050269 | Haemophilus influenzae Rd KW20 |
| BCAL3413 | DEG10050213 | Haemophilus influenzae Rd KW20 |
| BCAL2071 | DEG10050343 | Haemophilus influenzae Rd KW20 |
| BCAL2220 | DEG10050019 | Haemophilus influenzae Rd KW20 |
| BCAL1019 | DEG10130054 | Acinetobacter baylyi ADP1 |
| BCAL2853 | DEG10130054 | Acinetobacter baylyi ADP1 |
| BCAL2393 | DEG10180110 | Escherichia coli MG1655 I |
| BCAL0971 | DEG10150109 | Pseudomonas aeruginosa UCBPP-PA14 |
| BCAL2899 | DEG10150109 | Pseudomonas aeruginosa UCBPP-PA14 |
| BCAL3142 | DEG10180441 | Escherichia coli MG1655 I |
| BCAL1263 | DEG10080151 | Helicobacter pylori 26695 |
| BCAL2156 | DEG10050131 | Haemophilus influenzae Rd KW20 |
| BCAL3420 | DEG10190199 | Escherichia coli MG1655 II |
| BCAL0239a | DEG10120058 | Francisella novicida U112 |
| BCAL3338 | DEG10030355 | Vibrio cholerae N16961 |
| BCAL0492 | DEG10130090 | Acinetobacter baylyi ADP1 |
| BCAL1889 | DEG10020215 | Staphylococcus aureus N315 |
| BCAL2677 | DEG10030502 | Vibrio cholerae N16961 |
| BCAL0403 | DEG10050051 | Haemophilus influenzae Rd KW20 |
| BCAL1487 | DEG10030251 | Vibrio cholerae N16961 |
| BCAL1877 | DEG10110223 | Salmonella typhimurium LT2 |
| BCAL2340 | DEG10120377 | Francisella novicida U112 |
| BCAL1012 | DEG10100408 | Mycobacterium tuberculosis H37Rv |
| BCAL2858 | DEG10100408 | Mycobacterium tuberculosis H37Rv |
| BCAL0037 | DEG10180547 | Escherichia coli MG1655 I |
| BCAL0250 | DEG10130416 | Acinetobacter baylyi ADP1 |
| BCAL1864 | DEG10050079 | Haemophilus influenzae Rd KW20 |
| BCAL1983 | DEG10180217 | Escherichia coli MG1655 I |
| BCAL3337 | DEG10180312 | Escherichia coli MG1655 I |
| BCAL0909 | DEG10150070 | Pseudomonas aeruginosa UCBPP-PA14 |
| BCAL1966 | DEG10150242 | Pseudomonas aeruginosa UCBPP-PA14 |
| BCAL0247 | DEG10130419 | Acinetobacter baylyi ADP1 |
| BCAL2424 | DEG10050611 | Haemophilus influenzae Rd KW20 |
| BCAL2714 | DEG10150325 | Pseudomonas aeruginosa UCBPP-PA14 |
| BCAL2725 | DEG10150270 | Pseudomonas aeruginosa UCBPP-PA14 |
| BCAL0508 | DEG10160122 | Salmonella enterica serovar Typhi |
| BCAL3272 | DEG10030175 | Vibrio cholerae N16961 |
| BCAL3412 | DEG10050303 | Haemophilus influenzae Rd KW20 |
| BCAL2836 | DEG10050583 | Haemophilus influenzae Rd KW20 |
| BCAL2147 | DEG10160048 | Salmonella enterica serovar Typhi |
| BCAL3147 | DEG10130337 | Acinetobacter baylyi ADP1 |
| BCAL0742 | DEG10180534 | Escherichia coli MG1655 I |
| BCAL3441 | DEG10150274 | Pseudomonas aeruginosa UCBPP-PA14 |
| BCAL0995 | DEG10030390 | Vibrio cholerae N16961 |
| BCAL1554 | DEG10100556 | Mycobacterium tuberculosis H37Rv |
| BCAL2875 | DEG10030390 | Vibrio cholerae N16961 |
| BCAL0738 | DEG10110109 | Salmonella typhimurium LT2 |
| BCAL0745 | DEG10010086 | Bacillus subtilis 168 |
| BCAL1942 | DEG10030065 | Vibrio cholerae N16961 |
| BCAL3196 | DEG10020193 | Staphylococcus aureus N315 |
| BCAL2731 | DEG10030236 | Vibrio cholerae N16961 |
| BCAL1505 | DEG10030133 | Vibrio cholerae N16961 |
| BCAL1888 | DEG10030274 | Vibrio cholerae N16961 |
| BCAL0243 | DEG10150041 | Pseudomonas aeruginosa UCBPP-PA14 |
| BCAL1894 | DEG10080061 | Helicobacter pylori 26695 |
| BCAL3141 | DEG10160206 | Salmonella enterica serovar Typhi |
| BCAL3397 | DEG10050465 | Haemophilus influenzae Rd KW20 |
| BCAL3349 | DEG10030746 | Vibrio cholerae N16961 |
| BCAL1963 | DEG10130274 | Acinetobacter baylyi ADP1 |
| BCAL2195 | DEG10030161 | Vibrio cholerae N16961 |
| BCAL3054 | DEG10130490 | Acinetobacter baylyi ADP1 |
| BCAL3305 | DEG10120247 | Francisella novicida U112 |
| BCAL0236 | DEG10030535 | Vibrio cholerae N16961 |
| BCAL3369 | DEG10150027 | Pseudomonas aeruginosa UCBPP-PA14 |
| BCAL1943 | DEG10190290 | Escherichia coli MG1655 II |
| BCAL0224 | DEG10120337 | Francisella novicida U112 |
| BCAL0483 | DEG10150263 | Pseudomonas aeruginosa UCBPP-PA14 |
| BCAL0805 | DEG10140117 | Mycoplasma pulmonis UAB CTIP |
| BCAL0481 | DEG10050016 | Haemophilus influenzae Rd KW20 |
| BCAL3264 | DEG10010155 | Bacillus subtilis 168 |
| BCAL0332 | DEG10050525 | Haemophilus influenzae Rd KW20 |
| BCAL0025 | DEG10020299 | Staphylococcus aureus N315 |
| BCAL2344 | DEG10120381 | Francisella novicida U112 |
| BCAL0901 | DEG10120391 | Francisella novicida U112 |
| BCAL0033 | DEG10130025 | Acinetobacter baylyi ADP1 |
| BCAL0555 | DEG10050301 | Haemophilus influenzae Rd KW20 |
| BCAL1965 | DEG10080094 | Helicobacter pylori 26695 |
| BCAL2770 | DEG10120248 | Francisella novicida U112 |
| BCAL0227 | DEG10030052 | Vibrio cholerae N16961 |
| BCAL1262 | DEG10130344 | Acinetobacter baylyi ADP1 |
| BCAL3429 | DEG10150228 | Pseudomonas aeruginosa UCBPP-PA14 |
| BCAL1515 | DEG10150205 | Pseudomonas aeruginosa UCBPP-PA14 |
| BCAL1919 | DEG10110149 | Salmonella typhimurium LT2 |
| BCAL3453 | DEG10190021 | Escherichia coli MG1655 II |
| BCAL0825 | DEG10020067 | Staphylococcus aureus N315 |
| BCAL2017 | DEG10050452 | Haemophilus influenzae Rd KW20 |
| BCAL0231 | DEG10190231 | Escherichia coli MG1655 II |
| BCAL2957 | DEG10150117 | Pseudomonas aeruginosa UCBPP-PA14 |
| BCAL2739 | DEG10190231 | Escherichia coli MG1655 II |
| BCAL1416 | DEG10180418 | Escherichia coli MG1655 I |
| BCAL2724 | DEG10150271 | Pseudomonas aeruginosa UCBPP-PA14 |
| BCAL1994 | DEG10160164 | Salmonella enterica serovar Typhi |
| BCAL1448 | DEG10150075 | Pseudomonas aeruginosa UCBPP-PA14 |
| BCAM1944 | DEG10160025 | Salmonella enterica serovar Typhi |
| BCAL2409 | DEG10190041 | Escherichia coli MG1655 II |
| BCAL3373 | DEG10130395 | Acinetobacter baylyi ADP1 |
| BCAL0421 | DEG10160277 | Salmonella enterica serovar Typhi |
| BCAL2348 | DEG10180469 | Escherichia coli MG1655 I |
| BCAL3270 | DEG10160001 | Salmonella enterica serovar Typhi |
| BCAL3389 | DEG10050368 | Haemophilus influenzae Rd KW20 |
| BCAL1481 | DEG10130387 | Acinetobacter baylyi ADP1 |
| BCAL0034 | DEG10130026 | Acinetobacter baylyi ADP1 |
| BCAL2096 | DEG10190139 | Escherichia coli MG1655 II |
| BCAL1507 | DEG10150286 | Pseudomonas aeruginosa UCBPP-PA14 |
| BCAL1003 | DEG10130313 | Acinetobacter baylyi ADP1 |
| BCAL2867 | DEG10130313 | Acinetobacter baylyi ADP1 |
| BCAL2646 | DEG10050455 | Haemophilus influenzae Rd KW20 |
| BCAL0880 | DEG10130100 | Acinetobacter baylyi ADP1 |
| BCAL0024 | DEG10120264 | Francisella novicida U112 |
| BCAL0036 | DEG10130028 | Acinetobacter baylyi ADP1 |
| BCAL1267 | DEG10130342 | Acinetobacter baylyi ADP1 |
| BCAL2993 | DEG10050582 | Haemophilus influenzae Rd KW20 |
| BCAL2224 | DEG10130294 | Acinetobacter baylyi ADP1 |
| BCAM0746 | DEG10130167 | Acinetobacter baylyi ADP1 |
| BCAL2061 | DEG10050104 | Haemophilus influenzae Rd KW20 |
| BCAL2181 | DEG10150107 | Pseudomonas aeruginosa UCBPP-PA14 |
| BCAL3436 | DEG10150236 | Pseudomonas aeruginosa UCBPP-PA14 |
| BCAL3146 | DEG10160337 | Salmonella enterica serovar Typhi |
| BCAL2950 | DEG10030375 | Vibrio cholerae N16961 |
| BCAL0611 | DEG10150333 | Pseudomonas aeruginosa UCBPP-PA14 |
| BCAL3010 | DEG10190252 | Escherichia coli MG1655 II |
| BCAL0982 | DEG10160120 | Salmonella enterica serovar Typhi |
| BCAL2888 | DEG10160120 | Salmonella enterica serovar Typhi |
| BCAL2063 | DEG10130475 | Acinetobacter baylyi ADP1 |
| BCAL3336 | DEG10130291 | Acinetobacter baylyi ADP1 |
| BCAL2194 | DEG10050129 | Haemophilus influenzae Rd KW20 |
| BCAL2341 | DEG10120378 | Francisella novicida U112 |
| BCAL1468 | DEG10110052 | Salmonella typhimurium LT2 |
| BCAL3421 | DEG10150294 | Pseudomonas aeruginosa UCBPP-PA14 |
| BCAL2455 | DEG10030490 | Vibrio cholerae N16961 |
| BCAL1413 | DEG10030209 | Vibrio cholerae N16961 |
| BCAL3361 | DEG10150144 | Pseudomonas aeruginosa UCBPP-PA14 |
| BCAL1995 | DEG10110034 | Salmonella typhimurium LT2 |
| BCAL1901 | DEG10030039 | Vibrio cholerae N16961 |
| BCAL2198 | DEG10180388 | Escherichia coli MG1655 I |
| BCAL2454 | DEG10180449 | Escherichia coli MG1655 I |
| BCAL2207 | DEG10110014 | Salmonella typhimurium LT2 |
| BCAL0219 | DEG10180508 | Escherichia coli MG1655 I |
| BCAL0232 | DEG10180508 | Escherichia coli MG1655 I |
| BCAL2417 | DEG10190073 | Escherichia coli MG1655 II |
| BCAL0485 | DEG10150264 | Pseudomonas aeruginosa UCBPP-PA14 |
| BCAL1449 | DEG10050427 | Haemophilus influenzae Rd KW20 |
| BCAL1486 | DEG10190117 | Escherichia coli MG1655 II |
| BCAL2190 | DEG10110164 | Salmonella typhimurium LT2 |
| BCAL2179 | DEG10010237 | Bacillus subtilis 168 |
| BCAL2638 | DEG10130043 | Acinetobacter baylyi ADP1 |
| BCAL0145 | DEG10100518 | Mycobacterium tuberculosis H37Rv |
| BCAL3433 | DEG10160183 | Salmonella enterica serovar Typhi |
| BCAL3197 | DEG10150275 | Pseudomonas aeruginosa UCBPP-PA14 |
| BCAL3461 | DEG10150250 | Pseudomonas aeruginosa UCBPP-PA14 |
| BCAL3030 | DEG10190075 | Escherichia coli MG1655 II |
| BCAL2083 | DEG10150100 | Pseudomonas aeruginosa UCBPP-PA14 |
| BCAL2456 | DEG10050464 | Haemophilus influenzae Rd KW20 |
| BCAL1941 | DEG10030066 | Vibrio cholerae N16961 |
| BCAL0509 | DEG10190171 | Escherichia coli MG1655 II |
| BCAL1884 | DEG10120242 | Francisella novicida U112 |
| BCAL2339 | DEG10120376 | Francisella novicida U112 |
| BCAL3428 | DEG10150229 | Pseudomonas aeruginosa UCBPP-PA14 |
| BCAL2359 | DEG10100485 | Mycobacterium tuberculosis H37Rv |
| BCAL3370 | DEG10130093 | Acinetobacter baylyi ADP1 |
| BCAL3049 | DEG10130169 | Acinetobacter baylyi ADP1 |
| BCAL1873 | DEG10030539 | Vibrio cholerae N16961 |
| BCAL2146 | DEG10130174 | Acinetobacter baylyi ADP1 |
| BCAL3281 | DEG10050603 | Haemophilus influenzae Rd KW20 |
| BCAL2994 | DEG10150317 | Pseudomonas aeruginosa UCBPP-PA14 |
| BCAL2839 | DEG10150018 | Pseudomonas aeruginosa UCBPP-PA14 |
| BCAL0310 | DEG10130110 | Acinetobacter baylyi ADP1 |
| BCAL2389 | DEG10130292 | Acinetobacter baylyi ADP1 |
| BCAL0387 | DEG10180201 | Escherichia coli MG1655 I |
| BCAL0484 | DEG10130126 | Acinetobacter baylyi ADP1 |
| BCAL2333 | DEG10120370 | Francisella novicida U112 |
| BCAL3344 | DEG10120232 | Francisella novicida U112 |
| BCAL0876 | DEG10150313 | Pseudomonas aeruginosa UCBPP-PA14 |
| BCAL2338 | DEG10120375 | Francisella novicida U112 |
| BCAL3304 | DEG10050107 | Haemophilus influenzae Rd KW20 |
| BCAL0612 | DEG10190261 | Escherichia coli MG1655 II |
| BCAL0254 | DEG10030517 | Vibrio cholerae N16961 |
| BCAL0399 | DEG10130045 | Acinetobacter baylyi ADP1 |
| BCAL2357 | DEG10130393 | Acinetobacter baylyi ADP1 |
| BCAL0312 | DEG10130112 | Acinetobacter baylyi ADP1 |
| BCAL2841 | DEG10190170 | Escherichia coli MG1655 II |
| BCAL3416 | DEG10050055 | Haemophilus influenzae Rd KW20 |
| BCAL0482 | DEG10030073 | Vibrio cholerae N16961 |
| BCAL0903 | DEG10160288 | Salmonella enterica serovar Typhi |
| BCAL2150 | DEG10130212 | Acinetobacter baylyi ADP1 |
| BCAL3458 | DEG10150249 | Pseudomonas aeruginosa UCBPP-PA14 |
| BCAL3339 | DEG10160083 | Salmonella enterica serovar Typhi |
| BCAL3452 | DEG10130108 | Acinetobacter baylyi ADP1 |
| BCAL0869 | DEG10130192 | Acinetobacter baylyi ADP1 |
| BCAL0374 | DEG10120325 | Francisella novicida U112 |
| BCAL2126 | DEG10030428 | Vibrio cholerae N16961 |
| BCAL2109 | DEG10050571 | Haemophilus influenzae Rd KW20 |
| BCAL2676 | DEG10050625 | Haemophilus influenzae Rd KW20 |
| BCAL0904 | DEG10150003 | Pseudomonas aeruginosa UCBPP-PA14 |
| BCAL1269 | DEG10160225 | Salmonella enterica serovar Typhi |
| BCAL1883 | DEG10190144 | Escherichia coli MG1655 II |
| BCAL0479 | DEG10030196 | Vibrio cholerae N16961 |
| BCAL0041 | DEG10160267 | Salmonella enterica serovar Typhi |
| BCAL0800 | DEG10130356 | Acinetobacter baylyi ADP1 |
| BCAL2332 | DEG10120369 | Francisella novicida U112 |
| BCAL0493 | DEG10130091 | Acinetobacter baylyi ADP1 |
| BCAL1506 | DEG10190183 | Escherichia coli MG1655 II |
| BCAL2408 | DEG10190077 | Escherichia coli MG1655 II |
| BCAL0428 | DEG10180543 | Escherichia coli MG1655 I |
| BCAL1467 | DEG10050092 | Haemophilus influenzae Rd KW20 |
| BCAL1926 | DEG10130039 | Acinetobacter baylyi ADP1 |
| BCAL2148 | DEG10150105 | Pseudomonas aeruginosa UCBPP-PA14 |
| BCAL3306 | DEG10190047 | Escherichia coli MG1655 II |
| BCAL0956 | DEG10170153 | Staphylococcus aureus NCTC 8325 |
| BCAL0996 | DEG10120300 | Francisella novicida U112 |
| BCAL2874 | DEG10120300 | Francisella novicida U112 |
| BCAL0953 | DEG10160189 | Salmonella enterica serovar Typhi |
| BCAL0679 | DEG10170056 | Staphylococcus aureus NCTC 8325 |
| BCAL2101 | DEG10130316 | Acinetobacter baylyi ADP1 |
| BCAL0710 | DEG10150066 | Pseudomonas aeruginosa UCBPP-PA14 |
| BCAL2764 | DEG10110070 | Salmonella typhimurium LT2 |
| BCAL3035 | DEG10130147 | Acinetobacter baylyi ADP1 |
| BCAL0423 | DEG10190255 | Escherichia coli MG1655 II |
| BCAL1270 | DEG10110203 | Salmonella typhimurium LT2 |
| BCAL3351 | DEG10130166 | Acinetobacter baylyi ADP1 |
| BCAL1899 | DEG10190054 | Escherichia coli MG1655 II |
| BCAL3135 | DEG10050317 | Haemophilus influenzae Rd KW20 |
| BCAL0237 | DEG10030534 | Vibrio cholerae N16961 |
| BCAL2103 | DEG10130124 | Acinetobacter baylyi ADP1 |
| BCAL0558 | DEG10130266 | Acinetobacter baylyi ADP1 |
| BCAL2710 | DEG10130450 | Acinetobacter baylyi ADP1 |
| BCAL0613 | DEG10030284 | Vibrio cholerae N16961 |
| BCAL3468 | DEG10160011 | Salmonella enterica serovar Typhi |
| BCAL2955 | DEG10130326 | Acinetobacter baylyi ADP1 |
| BCAL0264 | DEG10130153 | Acinetobacter baylyi ADP1 |
| BCAL1485 | DEG10190118 | Escherichia coli MG1655 II |
| BCAL2390 | DEG10150007 | Pseudomonas aeruginosa UCBPP-PA14 |
| BCAL3329 | DEG10150145 | Pseudomonas aeruginosa UCBPP-PA14 |
| BCAL1517 | DEG20060091 | Homo sapiens |
| BCAL0375 | DEG10150280 | Pseudomonas aeruginosa UCBPP-PA14 |
| BCAL0260 | DEG10190205 | Escherichia coli MG1655 II |
| BCAL2208 | DEG10050441 | Haemophilus influenzae Rd KW20 |
| BCAL2935 | DEG10150133 | Pseudomonas aeruginosa UCBPP-PA14 |
| BCAL3455 | DEG10030474 | Vibrio cholerae N16961 |
| BCAL2085 | DEG10150099 | Pseudomonas aeruginosa UCBPP-PA14 |
| BCAL2767 | DEG10130195 | Acinetobacter baylyi ADP1 |
| BCAL2186 | DEG10150122 | Pseudomonas aeruginosa UCBPP-PA14 |
| BCAL3388 | DEG10100232 | Mycobacterium tuberculosis H37Rv |
| BCAL2838 | DEG10050633 | Haemophilus influenzae Rd KW20 |
| BCAL0318 | DEG10130468 | Acinetobacter baylyi ADP1 |
| BCAL0280 | DEG10130454 | Acinetobacter baylyi ADP1 |
| BCAL2167 | DEG10150232 | Pseudomonas aeruginosa UCBPP-PA14 |
| BCAL0460 | DEG10030715 | Vibrio cholerae N16961 |
| BCAL0992 | DEG10180178 | Escherichia coli MG1655 I |
| BCAL2878 | DEG10180178 | Escherichia coli MG1655 I |
| BCAL1516 | DEG10150204 | Pseudomonas aeruginosa UCBPP-PA14 |
| BCAL2934 | DEG10130329 | Acinetobacter baylyi ADP1 |
| BCAL0426 | DEG10120008 | Francisella novicida U112 |
| BCAL3467 | DEG10150255 | Pseudomonas aeruginosa UCBPP-PA14 |
| BCAL1625 | DEG10050204 | Haemophilus influenzae Rd KW20 |
| BCAL2726 | DEG10030025 | Vibrio cholerae N16961 |
| BCAL3440 | DEG10130308 | Acinetobacter baylyi ADP1 |
| BCAL0496 | DEG10130151 | Acinetobacter baylyi ADP1 |
| BCAL0462 | DEG10110077 | Salmonella typhimurium LT2 |
| BCAL2092 | DEG10120124 | Francisella novicida U112 |
| BCAL2337 | DEG10120374 | Francisella novicida U112 |
| BCAL0736 | DEG10020092 | Staphylococcus aureus N315 |
| BCAL0993 | DEG10030392 | Vibrio cholerae N16961 |
| BCAL2877 | DEG10030392 | Vibrio cholerae N16961 |
| BCAL0873 | DEG10150312 | Pseudomonas aeruginosa UCBPP-PA14 |
| BCAL2091 | DEG10150094 | Pseudomonas aeruginosa UCBPP-PA14 |
| BCAL2916 | DEG10050418 | Haemophilus influenzae Rd KW20 |
| BCAL3439 | DEG10130307 | Acinetobacter baylyi ADP1 |
| BCAL0401 | DEG10180517 | Escherichia coli MG1655 I |
| BCAL2768 | DEG10190272 | Escherichia coli MG1655 II |
| BCAL3050 | DEG10150057 | Pseudomonas aeruginosa UCBPP-PA14 |
| BCAL0240 | DEG10150038 | Pseudomonas aeruginosa UCBPP-PA14 |
| BCAL2078 | DEG10150103 | Pseudomonas aeruginosa UCBPP-PA14 |

Table shows to which organisms in DEG there are homologs among the 454 core genes of *Burkholderiales*.
