# Supplementary material for: High Confidence Prediction of Essential Genes in Burkholderia Cenocepacia
Source: PLoS One. 2012 Jun 29;7(6):e40064. doi: 10.1371/journal.pone.0040064 (PMC3386938; doi:10.1371/journal.pone.0040064)
Supplement: Table S4 — Novel essential genes. (DOC) [file pone.0040064.s007.doc]

**Table S4. Novel essential genes.**

| **Locus tag** | **Gene name** | **Product** |
| --- | --- | --- |
| BCAL0266 |  | putative cytochrome c4 |
| BCAL0303 |  | Putative membrane protein |
| BCAL0328 | *petA* | ubiquinol-cytochrome c reductase iron-sulfur subunit |
| BCAL0330 | *petC* | cytochrome c1 precursor |
| BCAL0390 |  | metallo peptidase, family M61 |
| BCAL0402 |  | Putative uncharacterized protein |
| BCAL0425 |  | hypothetical protein |
| BCAL0470 |  | Putative uncharacterized protein |
| BCAL0503 |  | putative cobalamin synthesis protein |
| BCAL0506 |  | Putative uncharacterized protein |
| BCAL0554 |  | 5-formyltetrahydrofolate cyclo-ligase family protein |
| BCAL0758 |  | putative cytochrome oxidase assembly protein |
| BCAL0804 |  | hypothetical protein |
| BCAL0816 |  | Putative exported protein |
| BCAL0818 |  | putative arabinose 5-phosphate isomerase |
| BCAL0870 |  | Putative oxidoreductase |
| BCAL0893 |  | phosphotransferase enzyme family protein |
| BCAL0958 |  | hypothetical protein |
| BCAL0968 |  | Putative uncharacterized protein |
| BCAL0980 | *mobA* | molybdopterin-guanine dinucleotide biosynthesis protein A |
| BCAL1010 | *nagZ2* | beta-hexosaminidase |
| BCAL1013 |  | Putative uncharacterized protein |
| BCAL1033 |  | Putative uncharacterized protein |
| BCAL1257 |  | putative gultathione hydrolase |
| BCAL1478 |  | Putative hydrolase |
| BCAL1488 |  | MerR family regulatory protein |
| BCAL1518 |  | AFG1-like ATPase |
| BCAL1614 |  | NUDIX hydrolase |
| BCAL1825 |  | hypothetical protein |
| BCAL1860 | *pbhF* | put. polyhydroxyalkanoate (PHA) synthesis regulatory protein |
| BCAL1863 | *phbC* | Poly-beta-hydroxybutyrate polymerase |
| BCAL1881 |  | putative lipoprotein |
| BCAL1882 |  | Putative uncharacterized protein |
| BCAL1886 |  | radical SAM superfamily protein |
| BCAL1897 | *recR* | recombination protein RecR |
| BCAL1906 |  | Putative membrane protein |
| BCAL1944 |  | putative primosomal replication protein |
| BCAL1970 |  | thioesterase superfamily protein |
| BCAL1981 |  | Putative uncharacterized protein |
| BCAL1989 |  | putative carbohydrate kinase |
| BCAL1992 |  | putative acyl-CoA thioesterase precursor |
| BCAL1997 | *tig* | trigger factor |
| BCAL2044 | *ldcA* | L,D-carboxypeptidase A |
| BCAL2075 |  | Putative uncharacterized protein |
| BCAL2076 |  | putative RNA methylase protein |
| BCAL2082 |  | chaperone protein Skp precursor |
| BCAL2097 |  | Putative uncharacterized protein |
| BCAL2119 |  | Universal stress protein family protein |
| BCAL2149 |  | HhH-GPD superfamily base excision DNA repair protein |
| BCAL2166 |  | Putative lipoprotein |
| BCAL2199 |  | putative transcriptional regulator protein |
| BCAL2206 | *phaP* | Phasin-like protein |
| BCAL2213 |  | Oligopeptidase A |
| BCAL2227 |  | Putative membrane protein |
| BCAL2328 |  | Putative uncharacterized protein |
| BCAL2392 |  | hypothetical protein |
| BCAL2420 |  | putative depolymerase/histone-like protein |
| BCAL2430 |  | putative ATPases |
| BCAL2618 |  | Putative uncharacterized protein |
| BCAL2641 |  | Putative ornithine decarboxylase |
| BCAL2642 | *dcd* | Deoxycytidine triphosphate deaminase |
| BCAL2758 | *xseA* | exodeoxyribonuclease VII large subunit |
| BCAL2769 |  | putative nucleotide-binding protein |
| BCAL2788 |  | flavin reductase family protein |
| BCAL2857 |  | Putative uncharacterized protein |
| BCAL2860 | *nagZ1* | beta-hexosaminidase |
| BCAL2890 | *mobA* | molybdopterin-guanine dinucleotide biosynthesis protein A |
| BCAL2941 |  | Putative exported transglycosylase |
| BCAL2952 | *aroA* | 3-phosphoshikimate 1-carboxyvinyltransferase |
| BCAL2958 | *ompA* | putative ompA family protein |
| BCAL2960 |  | putative phosphatase |
| BCAL3016 |  | coproporphyrinogen III oxidase |
| BCAL3057 |  | Putative lipoprotein |
| BCAL3132 | *rmlD* | dTDP-4-dehydrorhamnose reductase |
| BCAL3136 | *apaH* | diadenosine tetraphosphatase |
| BCAL3205 |  | Putative uncharacterized protein |
| BCAL3277 |  | putative RecN DNA repair protein |
| BCAL3290 | *glcF* | glycolate oxidase iron-sulfur subunit |
| BCAL3398 |  | Putative competence-damaged related protein |
| BCAL3400 | *pyrF* | Orotidine 5'-phosphate decarboxylase |
| BCAL3425 |  | putative sugar kinase |
| BCAL3432 |  | cytochrome c assembly protein |
| BCAL3456 |  | putative thioredoxin reductase |
| BCAM2216 |  | Putative uncharacterized protein |

Table shows 84 genes of the core genome of *Burkholderiales* which have not been found to be essential previously in any other organism.
